# CDK7-targeted therapy effectively disrupts cell cycle progression and oncogenic signaling in head and neck cancer

**DOI:** 10.1038/s41392-025-02452-z

**Published:** 2025-11-06

**Authors:** María Otero-Rosales, Miguel Álvarez-González, Irene Pazos, Beatriz de Luxán-Delgado, Sonia Del Marro, Esperanza Pozo-Agundo, Mar Rodríguez-Santamaría, Ana López-Fernández, Daniela Corte-Torres, Rocío Granda-Díaz, Saúl Álvarez-Teijeiro, Iván Fernández-Vega, Corina Lorz, Ramón García-Escudero, Juan P. Rodrigo, Konstantinos Tzelepis, George Vassiliou, Irene Ferrer, Mónica Álvarez-Fernández, Juana María García-Pedrero, Francisco Hermida-Prado

**Affiliations:** 1 Instituto de Investigación Sanitaria del Principado de Asturias (ISPA), Oviedo, Spain; 2 Instituto Universitario de Oncología del Principado de Asturias (IUOPA), University of Oviedo, Oviedo, Spain; 3 Spanish Biomedical Research Network in Cancer (CIBERONC), Instituto de Salud Carlos III, Madrid, Spain; 4 Targeted Therapies for Precisión Oncology Group and H12O-CNIO Lung Cancer Clinical Research Unit, Instituto de Investigación Sanitaria 12 de Octubre (imas12), CNIO, Madrid, Spain; 5 Molecular Oncology Unit, CIEMAT, Madrid, Spain; 6 Research Institute 12 de Octubre imas12, University Hospital 12 de Octubre, Madrid, Spain; 7 Molecular Histopathology Unit, Instituto Universitario de Oncología del Principado de Asturias (IUOPA), University of Oviedo, Oviedo, Spain; 8 Biobank of Principado de Asturias, Instituto de Investigación Sanitaria del Principado de Asturias (ISPA), Oviedo, Spain; 9 Department of Pathology, Hospital Universitario Central de Asturias, Oviedo, Spain; 10 Department of Otolaryngology, Hospital Universitario Central de Asturias, Oviedo, Spain; 11 Cambridge Stem Cell Institute, University of Cambridge, Cambridge, UK; 12 Department of Haematology, University of Cambridge, Cambridge, UK; 13 Milner Therapeutics Institute, University of Cambridge, Cambridge, UK; 14 Wellcome Sanger Institute, Wellcome Genome Campus, Cambridge, UK; 15 Centro de Investigación del Cáncer, CSIC-University of Salamanca, Campus Miguel de Unamuno, Salamanca, Spain

**Keywords:** Head and neck cancer, Cancer therapy

## Abstract

Head and neck squamous cell carcinoma (HNSCC) remains a prevalent and aggressive malignancy, characterized by a lack of targeted therapies and limited clinical benefits. Here, we conducted an optimized whole-genome CRISPR screen across five HNSCC cell lines aimed at identifying actionable genetic vulnerabilities for rapid preclinical evaluation as novel targeted therapies. Given their critical role in cancer, cyclin-dependent kinases (CDKs) were prioritized for further investigation. Among these, CDK7 was identified as an essential and targetable gene across all five cell lines, prompting its selection for in-depth functional and molecular characterization. Genetic and pharmacological inhibition of CDK7 significantly and consistently reduced tumor cell proliferation due to generalized cell cycle arrest and apoptosis induction. Additionally, CDK7 knockout (KO) and selective inhibitors (YKL-5-124 and samuraciclib) demonstrated potent antitumor activity, effectively suppressing tumor growth in HNSCC patient-derived organoids (PDOs), as well as in both cell line- and patient-derived xenograft (PDX) mouse models with minimal toxicity. Mechanistically, CDK7 inhibition led to a broad downregulation of gene sets related to cell cycle progression and DNA repair, and significantly reduced the transcription of essential genes and untargetable vulnerabilities identified by our CRISPR screen. These findings highlight CDK7 as a promising therapeutic target for HNSCC. Our study provides strong evidence of the robust antitumor activity of CDK7-selective inhibition in disease-relevant preclinical models, strongly supporting its progression to clinical testing.

## Introduction

Head and neck squamous cell carcinoma (HNSCC) is the sixth most prevalent cancer globally, with significant morbidity and mortality rates. Despite advances in multimodal treatment strategies, including surgery, radiation, and chemotherapy, the five-year survival rate for HNSCC patients remains between 40 and 50%.^1^ The prognosis is particularly unfavorable for patients with recurrent or metastatic disease.^2^ Nowadays, precision medicine for HNSCC is restricted to the EGFR-specific inhibitor cetuximab and the immunotherapy agents nivolumab and pembrolizumab.^3–5^ Treatment with cetuximab benefits a minority of HNSCC patients despite harboring high *EGFR* gene amplification, and its effectiveness is often hindered by tumor resistance. Similarly, even though immunotherapeutic agents such as nivolumab and pembrolizumab have recently emerged as promising treatment options, their clinical benefit in HNSCC patients is rather low (20–30%).

There is an unmet need for identifying novel therapeutic targets to improve treatment outcomes in this disease. Cyclin-dependent kinases (CDKs) are pivotal in cancer therapy due to their role in cell cycle regulation and proliferation. CDK4/6 inhibitors such as palbociclib have demonstrated efficacy, particularly in hormone receptor-positive breast cancer.^6–8^ However, their therapeutic capability/application is often limited by the development of drug resistance and adverse side effects. In HNSCC, CDK inhibitors hold promising therapeutic potential and continue to be an active area of research. Recent studies suggest a potential synergy when combining palbociclib with cetuximab and radiotherapy.^9^ Among the CDKs, CDK7 has been identified as a crucial regulator in cancer biology. This kinase forms a trimeric complex with Cyclin H and MAT1, functioning as a Cdk-activating kinase (CAK) that regulates both cell cycle progression and global transcription. CDK7 controls the cell cycle at different levels by phosphorylating CDKs 1, 2, 4, and 6 in their T-loops promoting their activation.^10–13^ Additionally, the CAK complex is a key component of the transcription factor TFIIH involved in transcription initiation and elongation.^14^ Furthermore, CDK7 is aberrantly overexpressed in various cancer types^15–17^ and significant progress has been made in developing selective CDK7 inhibitors. These inhibitors are currently demonstrating preclinical efficacy across multiple tumor types, including breast,^18,19^ pancreatic,^20^ and lung cancer^21^ among others.

Previous in vivo studies indicate that global CDK7 loss has minimal impact on non-proliferating tissues while predominantly impacting rapidly dividing cells,^22^ highlighting CDK7 inhibition as a promising therapeutic strategy for selectively targeting cancer cells with limited toxicity to normal tissues. For the last decade, genome-wide CRISPR screens have revolutionized the landscape of target identification in biomedical research, offering a powerful tool to elucidate the functional relevance of genes across the entire genome.^23,24^ While being extensively utilized in the past several years to define the landscape of important biological processes, these screens continue to be developed and are indispensable in uncovering novel therapeutic targets and dissecting complex biological pathways in vitro and in vivo.^25–30^

Taking advantage of an optimized genome-wide functional CRISPR screen,^28^ we aimed to identify actionable genetic vulnerabilities that can be rapidly evaluated as potential targeted therapies in preclinical HNSCC models. As a result, our study led to the identification of CDK7 as a promising clinically targetable vulnerability in HNSCC. A detailed functional and molecular characterization of available CDK7-selective inhibitors further contributed to unprecedentedly demonstrate their robust antitumor activity in a broad range of disease-relevant cellular, patient-derived organoids (PDOs) and animal models. These findings encourage future clinical testing in HNSCC patients.

## Results

### Genome-wide functional CRISPR-Cas9 screen reveals CDK7 as a vulnerability in HNSCC cells

**Fig. 1:**
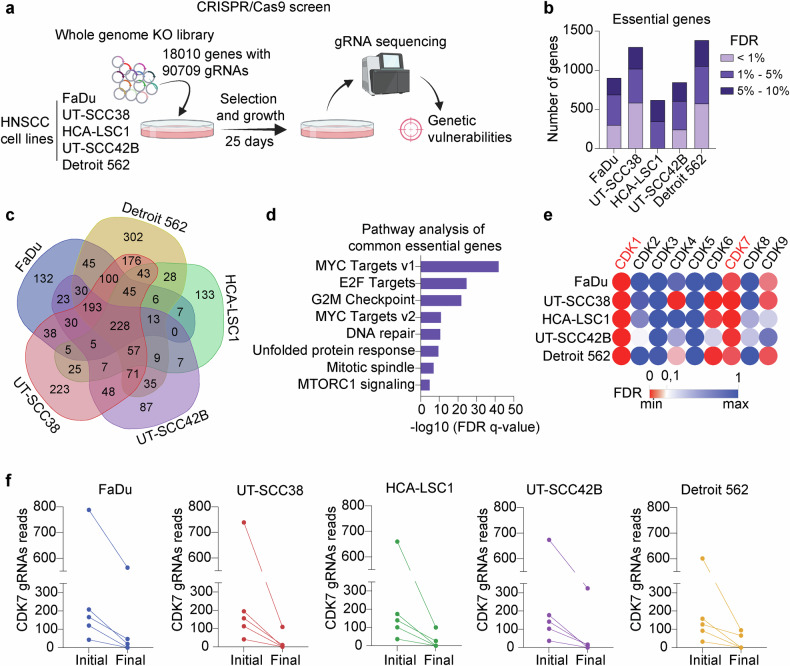
Whole-genome CRISPR screen uncovers genetic essentialities in HNSCC cell lines. **a** Schematic diagram depicting the experimental workflow of the functional CRISPR/Cas9 KO screen performed in a panel of five different HNSCC cell lines. Created in https://BioRender.com. **b** Bar graph showing the number of essential genes identified in each HNSCC cell line, categorized by FDR. **c** Venn diagram illustrating the overlap of essential genes across all five HNSCC cell lines tested (FDR < 0.10). **d** Pathway analysis of genes commonly essential across all HNSCC cells. **e** Heat map displaying the essentiality of genes coding for CDKs, based on FDR. **f** Line graph showing gRNAs counts of CDK7 gene at the initial (day 0) and final time points (day 25) of the screen for each cell line

In an attempt to uncover essential genes with therapeutic potential, we conducted a CRISPR-Cas9 knockout screen across five different HNSCC cell lines: FaDu, UT-SCC38, HCA-LSC1, UT-SCC42B and Detroit 562, which recapitulate the genetic and clinical diversity typically found in HNSCC (Supplementary Fig. 1a, b). We used a comprehensive whole-genome library, targeting 18,010 genes with 90,709 different gRNAs.^28^ The HNSCC cell lines were transduced using a low multiplicity of infection (MOI) of 0.3 and subsequently selected with puromycin to ensure efficient integration of the gRNAs. Cells were cultured for 25 days, after which genomic DNA was extracted, and gRNAs were amplified and sequenced (Fig. 1a and Supplementary Fig. 1c–e). Guide-RNA depletion and enrichment was analyzed using MAGeCK software^31^ to identify negatively and positively regulated genes, indicative of a role in cell viability.

Cell-line-specific and commonly essential genes were determined based on their false discovery rate (FDR) score. Only genes with FDR < 0.10 were considered for further analysis, thus identifying between 500 and 1500 essential genes per cell line (Fig. 1b). A focus on essential genes across multiple cell lines revealed 228 genes consistently essential in all five analyzed HNSCC cell lines (Fig. 1c and Supplementary Data 1), including well-known oncogenes and drivers of HNSCC cell proliferation such as MYC and CCND1. Pathway analysis of the common essential genes showed a significant enrichment in MYC and E2F targets and cell cycle checkpoint-related gene sets (Fig. 1d).

Given the translational potential of CDK inhibitors, we focused our attention on the essentiality of CDK proteins as targetable and druggable candidates. CDK1 and CDK7 emerged as the most crucial genes across all five HNSCC cell lines, while other CDKs such as CDK2 or CDK5 were found to be non-essential, or showed selectivity to certain cell lines, such as CDK4, CDK6 or CDK9 (Fig. 1e). CDK1 is the only CDK in mammals that is essential for cell cycle progression.^32^ However, to date, CDK1 inhibitors have been limited by high toxicity and insufficient clinical efficacy. Interestingly, CDK7-selective inhibitors have been recently developed and are under current active testing in clinical trials.^33,34^ Moreover, CPTAC-HNSCC data analysis showed that CDK7 protein levels significantly increased in tumors compared to normal tissue counterparts (Supplementary Fig. 1f) and DepMap data analysis demonstrated that HNSCC cell lines are among the most sensitive to CDK7 inhibition (Supplementary Fig. 1g). On this basis, we selected CDK7 for further investigation due to its critical role in cell cycle progression and transcription, as well as its function as an upstream regulator of other CDKs, including CDK1 itself. Single gRNA analysis revealed that every gRNA targeting CDK7 was depleted at the final time point in all HNSCC cell lines (Fig. 1f).

### Genetic validation of CDK7 essentiality in HNSCC cells

**Fig. 2:**
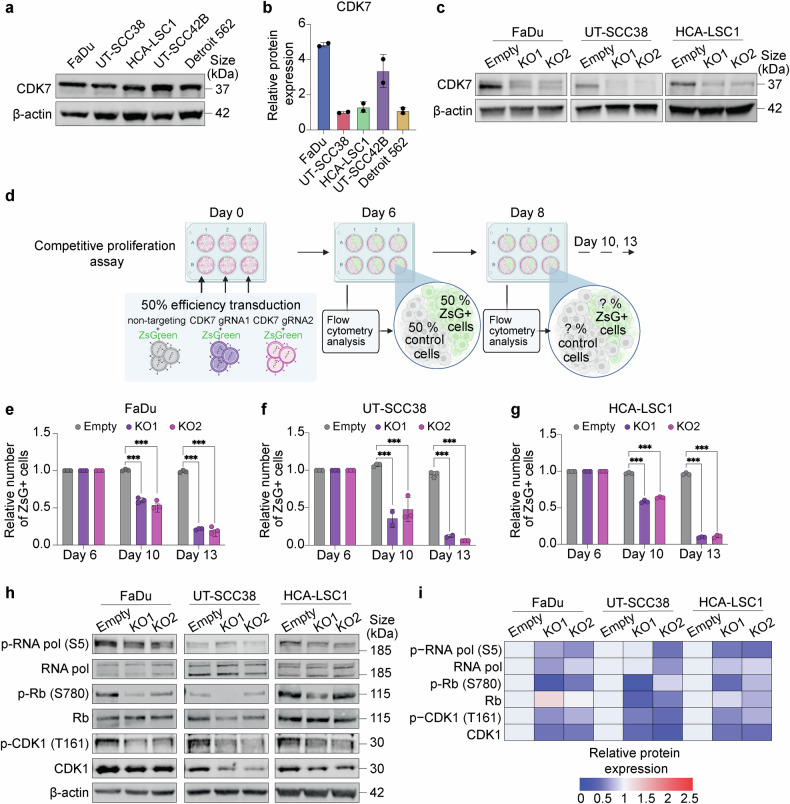
Genetic validation of CDK7 essentiality in HNSCC cells. **a** Western Blot analysis displaying CDK7 protein levels across our panel of HNSCC cell lines, with β-actin serving as the loading control. **b** Bar graph showing CDK7 protein levels quantified as fluorescent IRDye intensity across HNSCC cell lines, normalized to β-actin levels and relative to the lowest-expressing line (UT-SCC38). **c** Western Blot analysis of CDK7 expression levels in FaDu, UT-SCC38, and HCA-LSC1 cell lines transduced with two independent sgRNAs to knockout CDK7 gene (KO1 and KO2) or control cells transduced with the empty vector, and β-actin protein as the loading control. **d** Schematic diagram depicting the experimental workflow for the in vitro competitive proliferation assays. Created in https://BioRender.com. **e**–**g** Bar graphs showing the percentage of CDK7 KO cells harboring ZsG protein over different time points in FaDu (**e**), UT-SCC38 (**f**), and HCA-LSC1 (**g**) cell lines (***p < 0.001. Error bars, mean + SD of at least three replicates, Two-way ANOVA test). **h** Western blot analysis of the indicated CDK7 targets in empty vector- and CDK7 KO-transduced HNSCC cell lines, with β-actin protein serving as the loading control. **i** Heatmap illustrating the fluorescent IRDye quantification of the protein bands from (**h**) normalized to β-actin and relative to the empty control condition

To validate the essentiality of CDK7 as a candidate gene in our screen, we first analyzed CDK7 protein levels in our HNSCC cell line panel (Fig. 2a, b). We next performed targeted CRISPR knockouts of CDK7 (CDK7 KO) using two distinct single gRNAs in three HNSCC cell lines: FaDu, UT-SCC38 and HCA-LSC1, which harbored different endogenous levels of CDK7. We confirmed that these two gRNAs robustly reduced CDK7 protein levels in all three HNSCC cell lines (Fig. 2c). Subsequently, we carried out competitive co-culture assays between control and KO cells to validate the results of our screen (Fig. 2d). In these assays, CDK7 KO cells were significantly outcompeted by control cells in FaDu (Fig. 2e), UT-SCC38 (Fig. 2f) and HCA-LSC1 cells (Fig. 2g), thus confirming a significant reduction in cell viability upon CDK7 depletion. These results highlight the critical role of CDK7 in maintaining cell proliferation/survival and validate our initial findings from the CRISPR screen.

Even though comprehensive genomic analyses of the two selected CDK7-targeting gRNAs revealed no predicted high-risk off-target effects in coding regions (Supplementary Fig. 2a), we performed rescue experiments to further rule out potential non-specific effects. Specifically, we introduced a codon-optimized, CRISPR-resistant exogenous CDK7 construct designed to evade recognition by the targeting gRNAs, into three HNSCC cell lines from our panel. Western blot analysis confirmed efficient and selective depletion of endogenous CDK7 protein by both gRNAs across all three HNSCC cell lines, without affecting the exogenously expressed CDK7 (Supplementary Fig. 2b). Subsequently, competitive growth assays further demonstrated that exogenous expression of the codon-optimized CDK7 was effective and fully rescued the loss of cellular fitness caused by CDK7 knockout (Supplementary Fig. 2c–e). Together these results validate the specificity of our gRNAs and further support the essential role of CDK7 in HNSCC cell survival.

Furthermore, we assessed the impact of CDK7 KO on well-known downstream targets and cell cycle regulators. We found that CDK7 depletion led to reduced phosphorylation levels of the Carboxy-terminal domain (CTD) of RNA Polymerase II (Ser5), retinoblastoma protein (Rb) (Ser780), and CDK1 T-loop phosphorylation (Thr161) in the three HNSCC cell lines tested. Additionally, we observed a reduction in the total protein levels of Rb and CDK1 with the extent of these effects varying in a cell line-specific manner (Fig. 2h, i). These data demonstrate that CDK7 depletion effectively abrogates HNSCC cell proliferation by targeting several key downstream effectors and regulators of cell cycle control and transcription.

### Therapeutic potential of CDK7 pharmacological inhibition

**Fig. 3:**
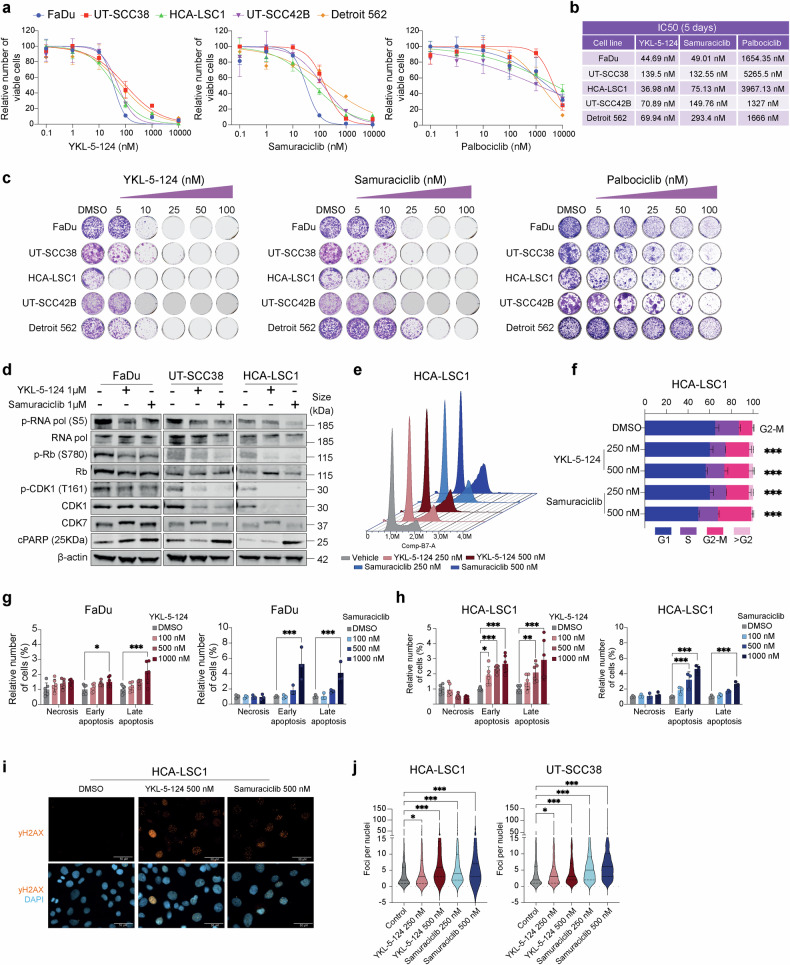
Functional characterization of CDK7 pharmacological inhibition in HNSCC cell lines. **a** Measurement of cell viability in our panel of five different HNSCC cell lines treated with increasing concentrations of the CDK7-selective inhibitors YKL-5-124 and samuraciclib, and compared to the CDK4/6 inhibitor palbociclib. Four replicates per condition. Data shown as average + SD. **b** Table summarizing average IC50 values for each cell line treated with the aforementioned compounds (*n* ≥ 2). **c** Colony formation assays with increasing concentrations of YKL-5-124, samuraciclib, and palbociclib in the indicated HNSCC cell lines. **d** Western Blot analysis of protein changes in the indicated CDK7 targets and cleaved PARP (cPARP) in three different HNSCC cell lines treated with either vehicle (DMSO), YKL-5-124 (1 μM), or samuraciclib (1 μM) for 24 h. β-actin protein was used as the loading control. **e** Flow cytometry analysis of cell cycle changes in HCA-LSC1 cells treated with the indicated doses of YKL-5-124 and samuraciclib for 48 h. Two replicates per condition. **f** Percentage of cells in each cell cycle phase shown in (**e**) are represented as a stacked barplot ± SD. Only significant accumulation of cells in a cell cycle phase is indicated. Statistical significance was calculated using two-way ANOVA Dunnett’s multiple comparisons test. ***p < 0.001. **g**, **h** Flow cytometry analysis of apoptosis and necrosis in the indicated HNSCC cell lines upon treatment for 48 h with increasing concentrations of either YKL-5-124 or samuraciclib. Two replicates per condition. Statistical significance was calculated using two-way ANOVA Dunnett’s multiple comparisons test. *p < 0.05; ***p < 0.001. **i**, **j** Analysis of γH2AX foci in HNSCC cells upon 48 h exposure to YKL-5-124 or samuraciclib by immunofluorescence (IF) microscopy. **i** Representative 20x images of HCA-LSC1 DAPI-stained nuclei (*in blue*) and γH2AX foci (in orange) (scale bar, 50 μm). **j** Violin plot showing γH2AX foci per nuclei in each condition. At least five field images were counted (≥400 nuclei). Statistical significance was calculated using two-way ANOVA Dunnett’s multiple comparisons test. *p < 0.05; ***p < 0.001

The robust effects of genetic CDK7 KO prompted us to evaluate the therapeutic potential of available CDK7-selective inhibitors in preclinical HNSCC models. We selected two distinct compounds: YKL-5-124, a selective covalent CDK7 inhibitor,^35^ and samuraciclib (CT7001, ICEC0942), an orally bioavailable, ATP-competitive inhibitor^36^ currently undergoing Phase I/II clinical trials in cancer patients.^33^ We first analyzed the effects of selective CDK7 inhibitors on the viability of a panel of five HNSCC cell lines. Both YKL-5-124 and samuraciclib (Fig. 3a) significantly reduced HNSCC cell viability, although lower IC50 values were observed for YKL-5-124 (ranging from 35 to 100 nM) than samuraciclib (range 30 to 200 nM) after 5 days of treatment (Fig. 3b).

Remarkably, both CDK7-selective inhibitors were more effective in decreasing HNSCC cell viability than the CDK4/6 inhibitor palbociclib (>10-fold higher IC50 values) (Fig. 3a, b). Analogous results were observed in colony formation assays performed over 14 days of treatment. Lower doses of YKL-5-124 and samuraciclib (ranging from 5 to 25 nM) completely abolished HNSCC cell proliferation, whereas considerably higher concentrations of palbociclib were required to achieve a comparable effect (Fig. 3c). Comparable results were obtained by testing two additional HNSCC cell lines derived from primary oral tumors (UT-SCC2 and Cal-33), showing similar IC50 values and anti-proliferative effects to the rest of our HNSCC panel from larynx and pharynx origin (Supplementary Fig. 3a, b).

We next investigated the immediate molecular effects of CDK7 inhibition on the phosphorylation of downstream protein substrates. HNSCC cells were treated with 1 µM of either YKL-5-124 or samuraciclib for 24 h to minimize potential compensatory mechanisms upon prolonged treatment. This resulted in decreased phosphorylation levels of CTD-RNA Polymerase II (Ser5), Rb (Ser780), and CDK1 (Thr161) in most HNSCC cell lines. Similarly, CDK7 inhibition resulted in decreased total CDK1 protein levels in a cell-line-dependent manner, mirroring the changes previously observed by CDK7 KO. CDK7 protein levels remained unchanged or slightly increased upon treatment with CDK7 inhibitors. Notably, a shift in CDK7 electrophoretic mobility was detected upon treatment with YKL-5-124 in all HNSCC, likely due to the covalent binding of this compound (Fig. 3d and Supplementary Fig. 3c–f).

The functional consequences of CDK7 pharmacological inhibition were also investigated on cell cycle dynamics and apoptosis. YKL-5-124 treatment led to cell accumulation in S and/or G2/M phases depending on the cell line analyzed, and samuraciclib treatment led to cell accumulation in G2/M phase in all five HNSCC cell lines (Fig. 3e, f and Supplementary Fig. 4a, b). This was also accompanied by a significant induction of apoptosis, especially after samuraciclib treatment, in all HNSCC cell lines tested (Fig. 3g, h and Supplementary Fig. 4c). Concordant to these findings, increased levels of cleaved PARP were also detected by western blot upon YKL-5-124 and samuraciclib treatment (Fig. 3d and Supplementary Fig. 3c–f). Concomitantly, selective CDK7 inhibition led to a significant increase in DNA damage, as indicated by elevated γH2AX levels in HNSCC cells treated with either YKL-5-124 or samuraciclib compared to control cells (Fig. 3i, j).

### Global transcriptional changes caused by CDK7 inhibition

**Fig. 4:**
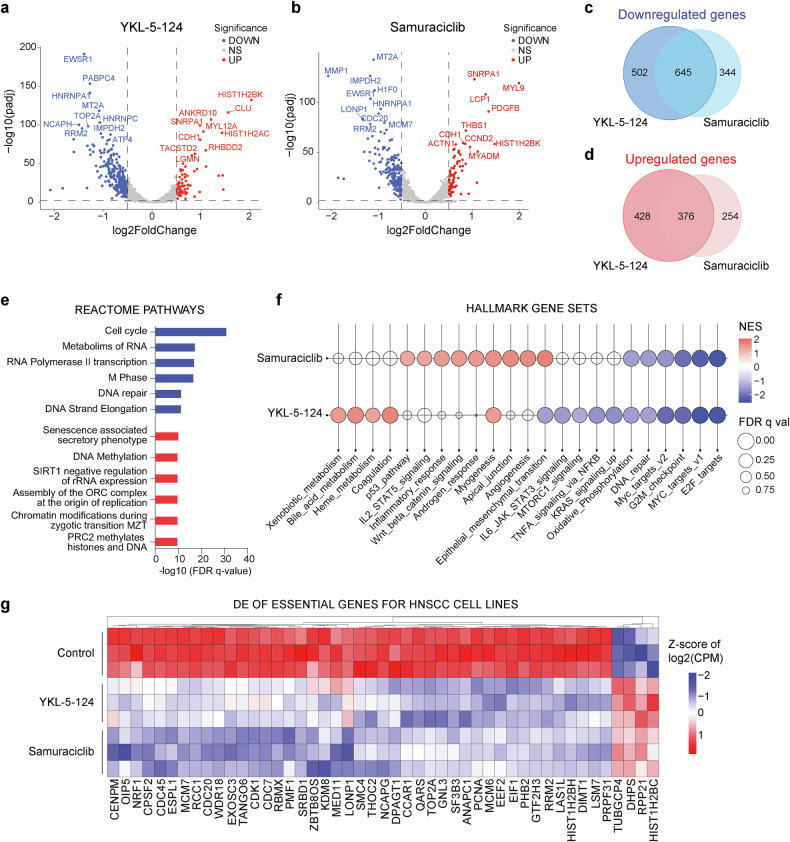
Global transcriptional changes and a common downregulated profile in FaDu cells caused by CDK7 inhibition. **a**, **b** Volcano plots illustrating differentially expressed genes after 48 h of treatment with either YKL-5-124 (**a**) or samuraciclib (**b**) at 250 nM (padj <0.05, log2fc < −0.5 or log2fc > 0.5). To enhance clarity, ribosomal coding genes were filtered out. **c**, **d** Venn diagram showing the unique and overlapping genes that were found downregulated (**c**) and upregulated (**d**) upon YKL-5-124 and samuraciclib treatment (padj<0.01, log2fc < −0.5 or log2fc > 0.5). **e** Reactome pathway analysis of the common downregulated (*blue bars*) and common upregulated (*red bars*) genes by both CDK7 inhibitors. **f** Balloon plot displaying Gene Set Enrichment Analysis of genes downregulated (*blue color*) or upregulated (*red color*) by either YKL-5-124 or samuraciclib. **g** Heatmap depicting the subsets of common essential genes identified by our CRISPR screen (FDR < 0.25) that were found commonly significantly upregulated (*in red*) or downregulated (*in blue*) by both CDK7 inhibitors, as compared to control DMSO-treated FaDu cells. Differential gene expression changes are shown as z-score of log2(CPM)

In order to delineate the global transcriptional changes caused by CDK7 inhibition, RNA-seq experiments were performed in FaDu cells treated with either YKL-5-124 or samuraciclib for 48 h. As represented by volcano plots, a total of 804 and 1,147 genes were found significantly up- and downregulated by YKL-5-124, respectively (Fig. 4a), and 630 and 989 genes were significantly up- and downregulated upon samuraciclib treatment, respectively (Fig. 4b). In addition, Venn diagrams depict the unique and overlapping changes by YKL-5-124 and samuraciclib, with a total of 645 common downregulated genes (Fig. 4c) and 376 common upregulated genes (Fig. 4d). GSEA using Reactome pathways database of common downregulated genes showed a predominance of gene sets related to cell cycle and mitotic pathways, as well as upregulation of specific gene sets by both compounds (Fig. 4e). GSEA of Hallmark gene sets further revealed a common downregulation of genes critical for cell cycle progression by both CDK7 inhibitors, including MYC and E2F targets, and G2/M checkpoint genes (Fig. 4f and Supplementary Fig. 5a). In addition, Supplementary Fig. 6 depicts gene expression changes across the cell cycle pathway upon treatment with CDK7 inhibitors. Moreover, genes involved in DNA repair were also found to be significantly and commonly downregulated by both compounds, suggesting a broader impact on cellular regulatory mechanisms essential for cell division and genome stability (Fig. 4f). Furthermore, YKL-5-124 treatment led to the downregulation of oncogenic pathways such as mTORC1, TNFα, and KRAS signaling, suggesting differential effects possibly due to varying mechanisms of action between these two CDK7 inhibitors (Supplementary Fig. 5b).

Interestingly, a number of essential genes identified in our genome-wide CRISPR screen (most of which undruggable targets) were significantly downregulated by CDK7 inhibition (Fig. 4g and Supplementary Data 2), suggesting a common downregulated profile of genetic vulnerabilities. According to these findings, CDK7 inhibition emerges as a promising therapeutic strategy to effectively and broadly target genetic vulnerabilities in HNSCC.

### Impact of CDK7 inhibition on mice xenograft models

**Fig. 5:**
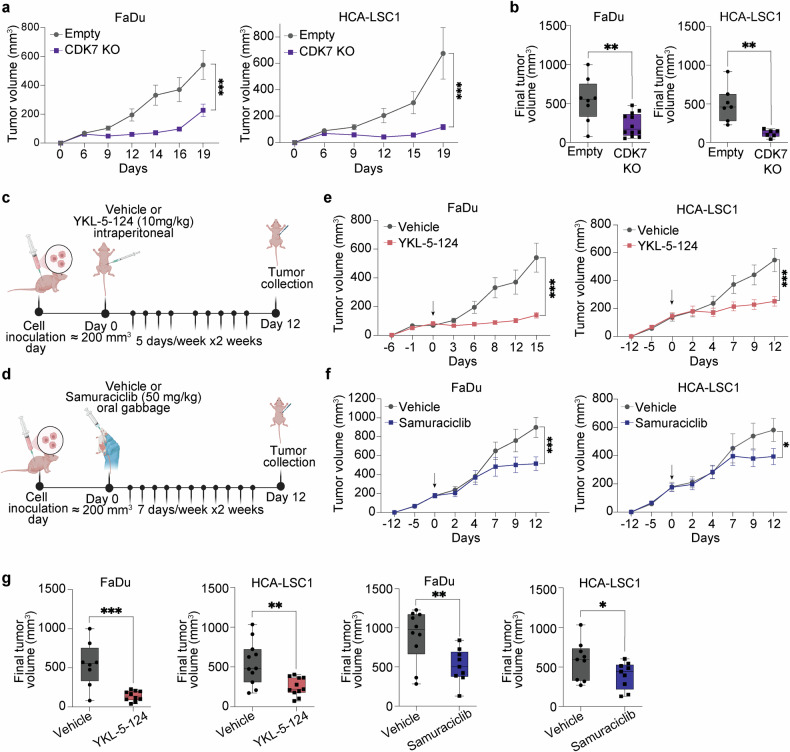
Impact of CDK7 inhibition on in vivo xenograft models. **a** Tumor growth of FaDu and HCA-LSC1 cell lines with CDK7 KO or empty vector (*n* = 12 tumors per group for FaDu xenografts, and *n* = 7 tumors per group for HCA-LSC1 xenografts). Data shown as mean ± SEM. Statistic two-way ANOVA, uncorrected Fisher’s LSD, ***p < 0.001. **b** Box plots representing final tumor volumes of CDK7 KO or empty vector groups. Unpaired t-test, ***p < 0.001. **c**, **d** Schematic representation of YKL-5-124 (**c**) and samuraciclib (**d**) testing in vivo. Created in https://BioRender.com. **e**, **f** Tumor growth of FaDu and HCA-LSC1 cell lines in presence of vehicle/YKL-5-124 (*n* = 12 tumors per treatment condition) (**e**) or vehicle/samuraciclib (*n* = 12 tumors per condition) (**f**) treatment for 12 days. Arrows indicate start of treatment. Data shown as mean ± SEM. Statistic two-way ANOVA, uncorrected Fisher’s LSD, ***p < 0.001. **g** Box plots representing final tumor volumes among the different experimental groups within pharmacological CDK7 inhibition experiments. Unpaired t-test, ***p < 0.001

We next examined the effects of both genetic and pharmacological inhibition of CDK7 on tumor growth in vivo. Deletion of CDK7 dramatically impaired the tumor growth of FaDu and HCA-LSC1 cell lines subcutaneously injected in immunodeficient mice (Fig. 5a), reaching final tumor volume reductions of 60 and 75% respectively (Fig. 5b). Noteworthy, treatment with YKL-5-124 and samuraciclib in a therapeutic setting (Fig. 5c, d) was also demonstrated to be effective at reducing the tumor volumes of both FaDu and HCA-LSC1 xenografts (Fig. 5e, f). Notably, YKL-5-124 exhibited a more potent antitumor activity, showing tumor reductions up to 74% after 12 days of treatment (Fig. 5g). By contrast, samuraciclib led to lower and delayed antitumor effects with tumor reductions of 40% after 12 days of treatment (Fig. 5g). Remarkably, none of these compounds caused any sign of adverse effects on the mice or body weight reduction (Supplementary Fig. 7).

### Impact of selective CDK7 inhibition on patient-derived xenograft

**Fig. 6:**
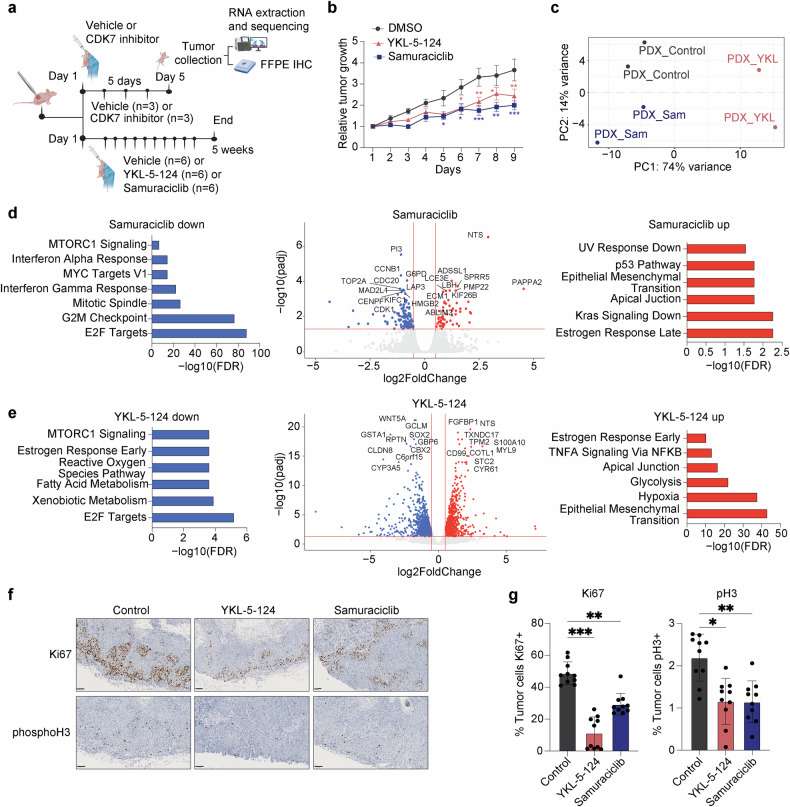
Impact of selective CDK7 inhibition in a HNSCC patient-derived xenograft (PDX) model. **a** Schematic representation of the experimental procedure performed in a HNSCC PDX model. Created in https://BioRender.com. **b** Tumor growth curves of PDX-bearing mice treated with vehicle (control), samuraciclib, or YKL-5-124. Data shown as mean ± SEM (*p < 0.05; **p < 0.01; ***p < 0.001, two-way ANOVA). **c** Principal component analysis (PCA) of RNA-seq data from mice PDX after 5 days of treatment. **d**, **e** Transcriptomic response to samuraciclib (**d**) and YKL-5-124 (**e**) treatment: volcano plot showing differentially expressed genes (*middle panel*), with Hallmark pathway enrichment of downregulated genes (*left side*) and upregulated genes (*right side*). **f** Representative immunohistochemistry images of Ki67 and phospho-histone H3 (pH3) staining in control- and CDK7 inhibitor-treated PDX tumors (scale bar, 100 μm). **g** QuPath quantification of Ki67- and pH3 as the percentage of positive cells in control and treated tumors. Data shown as mean ± SD (*p < 0.05; **p < 0.01; ***p < 0.001, two-way ANOVA)

To further validate the therapeutic potential of CDK7 inhibition in a more advanced preclinical HNSCC model, we assessed the efficacy of samuraciclib and YKL-5-124 in a patient-derived xenograft (PDX) model (Fig. 6a). CDK7 protein expression in the PDX model was confirmed by IHC (Supplementary Fig. 8a). Both inhibitors led to a significant reduction in tumor growth compared to vehicle-treated controls, thereby confirming their potent antitumor activity in this clinically relevant setting (Fig. 6b). Importantly, both treatments were well tolerated in mice. No signs of systemic toxicity were observed, indicated by normal hematological parameters (Supplementary Fig. 8b) as well as the absence of histopathological abnormalities in liver tissue (Supplementary Fig. 8c). These data collectively support the in vivo safety profile of CDK7 inhibition.

Global transcriptomic analysis of PDX-derived tumors after 5 days of treatment further demonstrated distinct gene expression profiles between treated and control groups, as shown by principal component analysis (Fig. 6c). Differential expression analysis identified a substantial number of upregulated and downregulated genes following both samuraciclib and YKL-5-124 treatment (Fig. 6d, e, *middle*). Samuraciclib treatment significantly downregulated gene sets involved in cell cycle regulation, DNA replication, and MYC targets (Fig. 6d, *left*), while upregulated genes were enriched in stress response, p53 signaling, inflammatory pathways, and epithelial-to-mesenchymal transition (EMT) (Fig. 6d, *right*). YKL-5-124 induced a comparable transcriptional response, including strong downregulation of E2F target genes and clear upregulation of EMT-associated programs (Fig. 6e). However, YKL-5-124 appears to cause more widespread expression changes probably reflecting tumor sample variability or compound-specific effects.

Immunohistochemical analysis of PDX-derived tumors further contributed to demonstrate a consistent significant reduction in the proliferation markers Ki67 and phospho-histone H3 (pH3) in tumors treated with either compound (Fig. 6f, g).

### Impact of selective CDK7 inhibition on patient-derived organoids

**Fig. 7:**
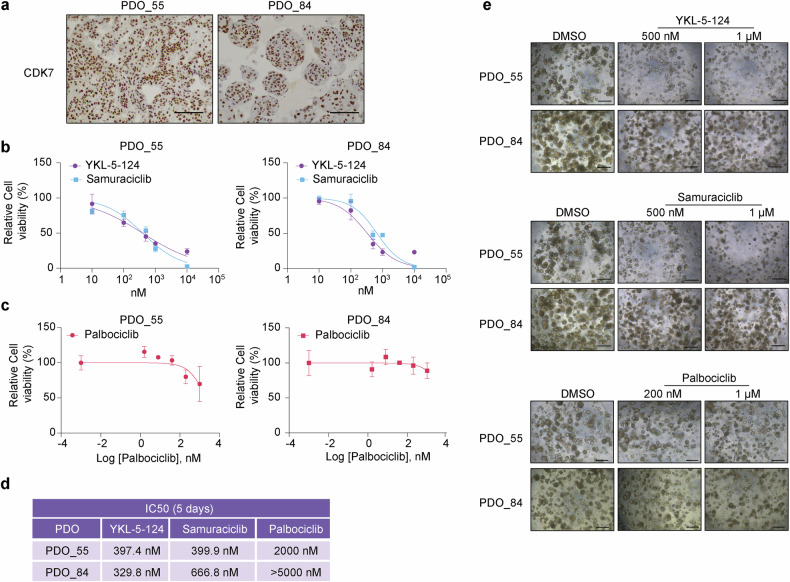
Therapeutic evaluation of CDK7-selective inhibitors in HNSCC PDOs. **a** Representative images of CDK7 expression in formed HNSCC PDOs analyzed by immunohistochemistry (scale bar, 100 μm). **b** Measurement of cell viability in HNSCC PDOs treated with increasing concentrations of YKL-5-124 and samuraciclib. Each condition was performed in quadruplicates. Data shown as mean ± SD. **c** Measurement of cell viability in HNSCC PDOs treated with increasing concentrations of palbociclib. Each condition was performed in triplicates. Data shown as mean ± SD. **d** Table summarizing IC50 values for each PDO treated with CDK7 inhibitors and palbociclib. **e** Representative images of formed HNSCC PDOs treated with the indicated doses of YKL-5-124, samuraciclib or palbociclib for five days (scale bar, 200 μm)

Finally, the therapeutic potential of CDK7 inhibitors was also tested in two HNSCC patient-derived organoid (PDO) models harboring endogenous CDK7 protein expression (Fig. 7a). Treatment of already formed PDOs with either YKL-5-124 or samuraciclib was effective in abrogating the growth of both HNSCC PDOs tested (Fig. 7b). The effects of CDK7-selective inhibitors were compared to those of the CDK4/6 inhibitor palbociclib (Fig. 7c). IC50s ranged from 329 to 397 nM for YKL-5-124 and 399 to 666 nM for samuraciclib, but were higher for palbociclib (Fig. 7d). Interestingly, PDO_84 showed resistance to palbociclib, while PDO_55 exhibited a much lower sensitivity to palbociclib compared to both CDK7 inhibitors (IC50 of 2 µM) (Fig. 7c, d). These data are in line with our results when testing these three compounds on the proliferation and viability of HNSCC cell lines (Fig. 3a–c). Representative images of PDO treatments are shown in Fig. 7e.

Also relevant from a therapeutic point of view, we explored the ability of CDK7-selective inhibitors to effectively target and eradicate cancer stem cells (CSC)-enriched HNSCC tumorsphere cultures. Clonal sphere-forming ability in non-adherent serum-free culture conditions is a hallmark of self-renewal and CSC-related phenotype. This is also the basis for the formation and growth of tumor organoids, which are 3D structures cultivated from patient-derived stem cells.^37^ Thus, we found that YKL-5-124 and samuraciclib were both effective in reducing the viability of CSC-enriched tumorsphere cultures in FaDu and UT-SCC38 cells, in a dose-dependent manner (Supplementary Fig. 9).

## Discussion

We performed a genome-wide CRISPR screen across five different HNSCC cell lines aimed to identify targetable genetic dependencies. Among all candidate essential genes identified, attention was focused on CDKs due to the central role of these kinases in cell cycle regulation and their unquestionable relevance in cancer therapy. Thus, a growing number of CDK-selective inhibitors are being extensively evaluated in clinical trials. In particular, three CDK4/6 inhibitors: palbociclib (Pfizer), abemaciclib (Eli Lilly), and ribociclib (Novartis), have been long approved for clinical use in combination with endocrine therapy in breast cancer^6–8^ and are under investigation for other solid tumors (NCT02465060, NCT03310879 and more). CDK4/6 inhibitors hold promise in HNSCC where *CDKN2A/p16* deletion and/or amplification of *CCND1* (encoding Cyclin D1) frequently occur, ultimately causing sustained CDK4/6 activation. However, our screening results show that CDK4 and CDK6 are only essential in certain HNSCC cell lines, aligned with their limited efficacy in early clinical trials.^38^ By contrast, CDK1 and CDK7 were revealed as widespread essentialities across all HNSCC cell lines tested, thereby emerging as the best targetable candidates. Even though CDK1 plays a critical role in cell cycle regulation, the clinical application of CDK1 inhibitors is hindered by their high toxicity and lack of efficacy in cancer patients.^39^

Notably, CDK7 functions as a CDK-activating kinase (CAK), regulating transcription and cell cycle progression through activation of multiple CDKs, including CDK1, CDK2, and CDK4/6. Therefore, selective CDK7 inhibition may offer a broader and more effective strategy to functionally target other essential CDKs, such as CDK1, and oncogenic programs in HNSCC. Several CDK7-selective inhibitors have proved efficacy in various preclinical models of breast,^18,19^ pancreatic,^20,40^ and lung cancer^21^ among others. Moreover, some CDK7 inhibitors have entered Phase I clinical trials for breast cancers.^33,34^ In the context of HNSCC, elevated levels of CDK7 have been associated with poor clinical outcomes.^41,42^ On this basis, CDK7 has been suggested as a potential therapeutic target for HNSCC; however, this possibility has not yet been explored in preclinical and/or clinical settings.

The present study provides the first broad-based evidence to comprehensively demonstrate the robust antitumor activity of CDK7-selective inhibitors using a wide range of disease-relevant cellular and animal models, including HNSCC cell lines, patient-derived organoids (PDOs), and patient-derived xenografts (PDXs). Both genetic and pharmacologic CDK7 inhibition consistently demonstrated potent antiproliferative effects in five different HNSCC cell lines, showing approximately 10-fold lower IC50 than the CDK4/6 inhibitor palbociclib. The dual action of YKL-5-124 and samuraciclib as cytostatic and cytotoxic agents, causing both cell cycle arrest and apoptosis induction, could plausibly explain the superior effectiveness to other exclusively cytostatic CDK inhibitors such as palbociclib.

At the molecular level, transcriptomic profiling of HNSCC cells and PDX models treated with YKL-5-124 and samuraciclib revealed significant and broad transcriptional changes, which might be due to impaired RNA polymerase II dependent transcriptional activity. Among those, downregulation of key cell cycle-related pathways, including MYC, E2F targets, and G2/M checkpoint regulators and DNA repair pathways. Accordingly, a significant accumulation of phosphorylated H2AX, a well-established marker of DNA damage, was observed upon CDK7 inhibition, further supporting a potential link between CDK7-mediated transcriptional blockade and compromised DNA repair. Moreover, CDK7 targeting resulted in decreased phosphorylation of CDK1 at T161 in its activation loop as well as total protein and mRNA levels, suggesting suppression of CDK1 activity. This is consistent with the cell cycle alterations, mostly G2 arrest, detected in CDK7-targeted cells and point to CDK7 inhibition as an effective strategy to block functionally the CDK7-CDK1 axis in HNSCC, observations that could probably be extrapolated to other tumor types.

In vivo, CDK7 inhibition demonstrated robust antitumor activity in both cell line-derived xenografts and patient-derived xenograft (PDX) models of HNSCC. Treatment with YKL-5-124 or samuraciclib led to significant tumor growth suppression, supporting both the efficacy and safety of CDK7-targeted therapy in clinically relevant models. Notably, none of these CDK7 inhibitors led to any noticeable adverse effects or blood/liver toxicity in mice, consistent with previous research demonstrating that CDK7 is non-essential in adult tissues with low proliferative rates and that CDK7 loss primarily impacts highly proliferative cells.^22^ Samuraciclib is currently undergoing Phase I/II clinical trials for various tumor types, in particular estrogen receptor-positive breast cancer where it also shows acceptable safety profile with evidence of antitumor activity in combination with endocrine therapy.^33^

The therapeutic potential of CDK7 inhibitors was also tested in already formed HNSCC PDOs, which offer a physiologically relevant patient-derived model that mimics the architectural and genetic heterogeneity of the primary tumors of origin. This led to concordant results further demonstrating that YKL-5-124 and samuraciclib were both highly effective at reducing the viability of HNSCC PDOs. Notably, CDK7 inhibition remained effective in palbociclib-resistant PDOs, reflecting the superior therapeutic efficacy of CDK7 inhibition and supporting its use in CDK4/6-refractory cases. In this sense, it is also worth mentioning that a recent study demonstrated CDK7 as a putative vulnerability to overcome CDK4/6 resistance in breast cancer.^19^

Tumor heterogeneity is a well-known feature that greatly complicates cancer treatment and a long-lasting clinical effectiveness. Research evidence indicates that heterogeneity develops through time as tumor-initiating stem cells, also known as CSCs.^43^ Hence, complete eradication of tumors requires therapies able to effectively eliminate the CSC subpopulations responsible for treatment resistance, relapse and metastasis.^44,45^ On this basis, it is a relevant finding that both YKL-5-124 and samuraciclib effectively reduced the viability of CSC-enriched tumorspheres as well as HNSCC PDOs, thereby suggesting that selective CDK7 targeting could also be an efficacious strategy to target and eradicate the CSC niche in HNSCC.

The present study uncovered a catalog of genetic vulnerabilities that could serve as the basis to design novel molecular-targeted treatments for HNSCC. As a proof-of-principle, CDK7 was revealed as a common essentiality in all HNSCC cell lines screened, and all concordantly displayed an exceptional dependency on CDK7 activity/function. Furthermore, genetic and pharmacologic CDK7 inhibition showed potent antitumor activity and efficacy in a wide range of disease-relevant HNSCC models. According to our findings, CDK7 inhibition emerges as a safe and feasible therapeutic strategy to effectively and broadly target CDK signaling and oncogenic pathways in HNSCC. All the herein preclinical results provide unprecedented support for future clinical testing of CDK7-selective inhibitors in HNSCC patients, ultimately helping to fill the gap of lack of effective molecular-targeted treatments for this disease.

## Materials and methods

### Ethics approval and consent to participate

This study was performed in accordance to the principles of the Declaration of Helsinki with the appropriate approval of the Ethical and Scientific Committees of the Hospital Universitario Central de Asturias and the Regional Ethics Committee from the Principality of Asturias (CEImPA) (date of approval 9 March 2023; approval number: 2023.018, for the project PI22/00167). Patient informed consent was obtained for all the tissue samples collected by the Biobank of Principado de Asturias (PT20/00161 and PT23/00077).

All animal experiments were performed in accordance with institutional guidelines for the care and use of laboratory animals. Experimental procedures were approved by the Animal Research Ethics Committees of the University of Oviedo, CIEMAT, Instituto de Salud Carlos III, and CNIO, under the following approvals: PROAE 46/2019 (1 August 2019), PROAE 03/2024 (8 March 2024), PROEX 045.8/21 (12 February 2021), and PROEX 073.3/24 (7 November 2024). Additional authorization was granted under Am. 001_IACUC.003-2024 (CBA 05_2024) on 4 March 2024.

### Cell lines and cell culture

FaDu (male, hypopharyngeal squamous cell carcinoma, grade II) and Detroit 562 cells (female, oropharyngeal squamous cell carcinoma, metastatic) were purchased from the ATCC. The HNSCC cell lines UT-SCC38 (male, laryngeal squamous cell carcinoma, T2N0M0, primary), UT-SCC42B (male, laryngeal squamous cell carcinoma, T4N3M0, metastatic) derived from laryngeal squamous carcinomas and UT-SCC2 (male, oral squamous cell carcinoma, T4N1M0) were kindly provided by Prof. Reidar Grenman (Department of Otolaryngology, University Central Hospital, Turku, Finland). Cal-33 (male, tongue squamous cell carcinoma) cell line was kindly provided by Prof. Silvio Gutkind (University of California San Diego, United States). HCA-LSC1 cell line was established in our laboratory from a male patient primary laryngeal squamous carcinoma chemotherapy-resistant.

Cells were grown in DMEM supplemented with 10% fetal bovine serum (FBS), 100 U/mL penicillin, 200 mg/mL streptomycin, 2 mmol/L L-glutamine, 20 mmol/L HEPES (pH 7.3), and 100 mmol/L non-essential amino acids. All cells derived from HPV-negative primary HNSCC. All cell lines were periodically tested for mycoplasma contamination by PCR using the Biotools Detection kit (Madrid, Spain) specifically amplifying a conserved region of the mycoplasma 16S RNA gene. Cell line authentication was carried out by DNA (STR) profiling at the SCT Core Facilities (University of Oviedo, Spain).

Cas9-expressing HNSCC cell lines were generated by lentiviral transduction using pKLV2-EF1a-Bsd2Cas9-W (Addgene, #67978). Blasticidin selection was initiated 3 days after transduction at 20 μg/mL and maintained for at least 14 days.

Exogenous CDK7-expressing cell lines were generated from Cas9-expressing HNSCC cell lines using the lentiviral vector, pLV[Exp]-mCherry:T2A:Hygro-EF1A > hCDK7[NM_001799.4](co)*/HA (Vector ID: VB250723-1187hyu), constructed and packaged by VectorBuilder. Hygromycin selection was initiated 3 days after transduction at 150-200 μg/mL and maintained for at least 6 days to ensure stable integration.

### Lentiviral production

For lentiviral production, HEK293T cells were co-transfected with the packaging plasmids pLP1 and pLP2, the envelope plasmid pLP/VSV-G (Life Technologies), and the plasmid or library of interest. Viral supernatants were harvested and used to transduce HNSCC cells, which were subsequently cultured in antibiotic-containing selection medium (puromycin, 2–3 μg/mL; or hygromycin, 150–200 μg/mL) for at least five days.

### CRISPR/Cas9 KO screen

CRISPR screens were conducted at the Wellcome Sanger Institute using the Human Improved Genome-wide Knockout CRISPR Library v1, a gift from Dr. Kosuke Yusa (Addgene #67989). This library includes 90,709 sgRNAs targeting 18,010 human genes, with approximately 6 sgRNAs per gene. Library representation at the time of transduction was maintained at 300-fold. For each HNSCC cell line, a total of 1.0 × 10^8^ cells were transduced with a predetermined volume of the genome-wide gRNA lentiviral supernatant that gave rise to 30% transduction efficiency. Two days after transduction, cells were selected with puromycin for 5 days and further cultured, always keeping the total population above 3.0 × 10^7^. After 25 days of culturing, at least 3.0 × 10^7^ cells were collected as the final time point.

### Illumina sequencing of gRNAs and statistical analysis

Genomic DNA extraction and Illumina sequencing of gRNAs were conducted as described previously.^24^ The numbers of reads for each guide were counted with an in-house script. Enrichment and depletion of guides and genes were analyzed using MAGeCK statistical package^31^ by comparing read counts from each cell line with counts from matching plasmid as the initial population and used for DNA extraction and gRNA sequencing.

### Generation of CDK7 KO cell lines

HNSCC cell lines expressing Cas9 were transduced with two specific CDK7 gRNAs (KO1 or KO2) by subcloning each gRNA targeting sequence (CDK7_KO1 guide RNA: TTCCATAAAATCAAAGACA; CDK7_KO2 guide RNA: TAAAAACCTTACCCTATGT) into the expression vector pKLV2-U6gRNA5(BbsI)-PKGpuro2AZsG-W (Addgene #67975) or with the empty vector as control and then, cells were selected in medium containing puromycin (2–3 μg/ml) for 6 days.

### Competitive proliferation assay

Cas9-expressing HNSCC cells were mixed after transduction and selection at a 1:1 ratio or transduced at 50% efficiency with lentiviral particles carrying plasmids encoding either a specific CDK7-targeting gRNA (CDK7 KO1 or KO2) or a non-targeting control plasmid all co-expressing ZsGreen (ZsG). The percentage of (ZsG)-positive cells vs non-fluorescent cells was measured by flow cytometry between days 6 and 13 post-transduction and normalized to the percentage of ZsG-positive cells at day 6. Data are represented as the relative number of ZsG-positive cells in each well.

### Drugs

YKL-5-124 and samuraciclib (CT7001) were obtained from MedChem Express. Palbociclib was obtained from Selleckchem. For in vitro studies, stock solutions of both compounds were prepared at a concentration of 10 mM in sterile dimethyl sulfoxide (DMSO) and stored at −80 °C. Prior to each experiment, the drugs were thawed and diluted to the desired final concentrations. DMSO was used as the vehicle control condition.

For in vivo studies, YKL-5-124 and samuraciclib were prepared in a vehicle of 10% DMSO, 40% PEG-300, 5% Tween-80, and 45% saline at a stock concentration of 20 mg/mL or 100 mg/mL, respectively, and stored at −80 °C. Dosing solutions were freshly prepared daily before administration. YKL-5-124 was administered intraperitoneally at 10 mg/kg, five days a week, with a corresponding intraperitoneal vehicle control group. Samuraciclib was given orally at 50 mg/kg daily for cell line-derived xenografts and 70 mg/kg 5 days a week for PDX model, with a separate oral vehicle control group.

### Cell viability assays

HNSCC cells were seeded into 96-well culture plates at a density of 2000–4000 cells per well and incubated overnight. Drugs were serially diluted in medium over a range of concentrations and added to the cells. After 5 days of treatment, cell viability was measured in triplicates or quadruplicates using MTS assay (CellTiter 96 Aqueous One Solution Cell Proliferation Assay from Promega, Madison, WI, USA) reading absorbance at 490 nm using a Synergy HT plate reader (BioTek, Winooski, VT, USA). For the IC50 studies, the number of viable cells upon each drug treatment was normalized to the number of vehicle (DMSO)-treated cells at day 5 and the IC50 values were calculated using GraphPad Prism10 as the [inhibitor] vs. normalized response – Variable slope function.

### Proliferation assays

HNSCC cells were seeded into 6-well culture plates at a density of 3000–5000 cells per well and incubated overnight. YKL-5-124 or samuraciclib were serially diluted in medium over a range of concentrations and added to the cells. Treatment was renewed every 3 days. After 14 days of culture, cells were fixed with methanol and stained with crystal violet 0.1% w/v. Colonies were then scanned with GS-800 Calibrated Imagen Densitometer (Bio-Rad, 170-7980) and images were analyzed with Fiji software to measure the colony-covered area per well. Data were normalized to the DMSO-treated control condition.

### Cell cycle analysis

HNSCC cells were plated in 6-well plates in complete cell culture medium. After 24 h, cells were treated with either YKL-5-124, samuraciclib or vehicle in growth media for 72 h. Then, cells were collected and fixed in cold 70% ethanol for at least 24 h at −20 °C. Cell cycle analysis was performed using FxCycle™ PI/RNase Staining Solution (Life Technologies, #F10797) to measure DNA content by flow cytometry, according to the manufacturer’s instructions. Cell percentage in each cell cycle phase was determined using FlowJo software’s Cell cycle algorithm. Only statistically significant changes in cell cycle phase distribution are reported.

### Apoptosis assay

HNSCC cells were plated in 6-well plates, incubated for 24 h, and then treated with either YKL-5-124, samuraciclib or vehicle for 48 h. Apoptotic cells were quantified through Annexin V and Propidium Iodide (PI) staining, using Dead Cell Apoptosis Kit with Annexin V FITC/Alexa Fluor™ 488 & Propidium Iodide for Flow Cytometry (Invitrogen, #V13242 and #V13241) according to the manufacturer’s instructions.

### Tumorsphere formation assays

HNSCC-derived cell lines were plated at a density of 500 cells/mL in 6-well tissue culture plates treated with a sterile solution of polyHEMA (10 g/L in 95% ethanol, Sigma) to prevent cell attachment. Cells were grown in DMEM-F12 (GE Healthcare) supplemented with 1% Glutamax and 2% B27 Supplement (Life Technologies), 10 ng/mL human bFGF and 20 ng/mL human EGF (PeproTech) and 100 U/mL penicillin and 200 mg/mL streptomycin (Thermo Scientific). In addition, fresh aliquots of EGF and bFGF were added every three days. After 7 days, tumorspheres were treated for 5 days with different concentrations of either YKL-5-124, samuraciclib, or DMSO as vehicle condition. Tumorsphere viability was measured using the CellTiter-Glo 3D assay (Promega, #G9681) and luminescence quantification using a Synergy HT plate reader (BioTek).

### Western blot analysis

Cells were lysed in RIPA buffer (Thermo Scientific, 89900) supplemented with protease and phosphatase inhibitors (Sigma Aldrich, 78430). Protein concentration was determined by BCA assay (Thermo Scientific, 23225), samples were separated to SDS-PAGE by using NuPAGE™ 4 to 12% Bis-Tris gels (Life Technologies) in MOPS running buffer and transferred to Trans-Blot® Turbo™ Midi Nitrocellulose membranes (Bio-Rad Laboratories). Membranes were blocked using 5% BSA in TBS-T for 1 h at room temperature. Membranes were then incubated with primary antibodies (Supplementary Table 1) overnight at 4 °C, then washed in TBS-T and incubated with secondary antibodies goat anti-rabbit IRDye 800CW or anti-mouse IRDye 680RD (IRDye, LICOR, at 1:10,000 dilution) for 1 h at room temperature. Fluorescence was measured using Odyssey® Fc Imager (LICOR Biosciences) and analyzed with Image Studio Lite software (LICOR Biosciences).

### Immunofluorescence staining

Cells were cultured on top of fibronectin-coated coverslips (1 μg/mL in PBS, for 30 min), and treated for 48 h with CDK7 inhibitors. Cells were then fixed in 4% paraformaldehyde for 10 min at room temperature. After two washes with PBS, cells were permeabilized with 0.5% Triton X-100 in PBS for 10 min at room temperature. After washing with PBS, cells were blocked with 3% BSA in PBS-Tween 1% for 30 min at room temperature and then incubated overnight at 4 °C with the primary antibody γH2AX (Ser139, Merck Millipore #05-636) at 1:250 dilution. Next day, primary antibody was washed four times with PBS-T and incubated at room temperature for 1 h with the secondary antibody Alexa Fluor 555 (Invitrogen #A21428) at 1:500 and, thereafter, washed off four times with PBS-T. Cell nuclei were then stained with DAPI (100 ng/mL in PBS) (ThermoFisher, #D1306) for 10 min. After washing two times with PBS, coverslips were mounted on microscope slides using mounting media (Dako Fluorescence Mounting Medium, Agilent, #S3023). Images were acquired with Zeiss Cell Observer Live Imaging (Zeiss, Thornwood, NY) and analyzed by FIJI software (NIH).

### RNA extraction, sequencing and bioinformatic analysis

Total RNA was extracted from pre-confluent FaDu cells using the GeneJET RNA Purification Kit (Thermo Scientific), with three biological replicates per condition. For PDX samples, RNA was isolated from frozen tumor tissues using the RNeasy Mini Kit (Qiagen, #74104), following the manufacturer’s instructions. RNA quantity and quality were assessed using the Qubit™ 4 Fluorometer (Invitrogen, #Q33226) and the Broad Range RNA Assay Kit (Invitrogen, #Q10211).

All steps following RNA extraction, including mRNA enrichment, library preparation, sequencing, and primary bioinformatic analysis, were performed by Novogene, Inc. (Cambridge, UK). Briefly, mRNA was purified using poly-T oligo-attached magnetic beads, fragmented, and reverse-transcribed to cDNA. Paired-end sequencing reads were aligned to the human reference genome (GRCh38) using Hisat2 v2.0.5, and gene expression was quantified using annotations from the GENCODE database. Differential expression analysis between CDK7 inhibitor-treated and control samples (three biological replicates per group) was carried out using the DESeq2 R package (v1.20.0).

Gene Set Enrichment Analysis (GSEA) was performed in-house using GSEA_4.1.0 on normalized gene count data. Hallmark and Reactome gene sets were used to identify enriched biological pathways. GSEA was also used to analyze commonly upregulated and downregulated genes upon CDK7 inhibition.

Expression changes of common essential genes following CDK7 inhibition were visualized as z-scores of log2 counts per million (CPM) values.

### Mouse xenografts

All experimental protocols were performed in accordance with the institutional guidelines of the University of Oviedo and CIEMAT, and approved by the corresponding Animal Research Ethical Committee prior to the study (date of approval 1 August 2019; approval number PROAE 46/2019; date of approval 8 March 2024; approval number PROAE 03/2024; date of approval 12 February 2021; approval number PROEX 045.8/21).

Female Athymic Nude-Fox1nu mice of 5–6 weeks old (ENVIGO RMS) were subcutaneously (s.c.) inoculated in the flanks with 1.5 × 10^6^ FaDu or 2 × 10^6^ HCA-LSC1 cells in culture medium mixed 1:2 with VitroGel® Hydrogel Matrix (The Well Bioscience, #VHM01). Once tumors reached a measurable size (between 100 and 200 mm^3^), mice were randomized into four treatment groups (six mice per group): (i) Intraperitoneal vehicle; (ii) Oral vehicle; (iii) YKL-5-124 (10 mg/kg, 5 doses/week, intraperitoneal) and (iv) samuraciclib (50 mg/kg, 7 doses/week, orally). Mice were monitored daily for signs of toxicity and tumor size was measured with a caliper 2–3 times a week.

Tumor volume was determined using the equation (*D* × *d*^2^)/6 × 3.14, where *D* is the maximum diameter, and *d* is the minimum diameter. Tumor volumes for all mice in each xenograft-treatment group were averaged to obtain the mean tumor volume for the corresponding group. Animals were sacrificed by CO_2_ asphyxiation or cervical dislocation when the tumors of the control group reached approximately 1000 mm^3^. Tumors were resected from the flanks, fixed in 4% paraformaldehyde and embedded in paraffin for histological evaluation.

### Patient-derived organoids (PDOs)

HNSCC PDOs were generated from fresh primary tumor biopsies from HNSCC patients surgically treated at the Hospital Universitario Central de Asturias (HUCA), following institutional review board guidelines, and approved by the Regional Ethics Committee of the Principality of Asturias (CEImPA) (date of approval 25 January 2021; approval number 2021.002, for the project PID2020-117236RB-100). Informed consent was obtained from all patients. Tissue samples were obtained through the Biobank of the Principality of Asturias (BioPA) (National Registry of Biobanks B.0000827) (PT23/00077 funded by ISCIII and co-funded by the European Union) and processed as described by Driehuis and colleagues.^46^

PDOs were grown in Matrigel droplets (Corning #356231) in expansion medium, consisting of basal medium (Advanced DMEM/F12 supplemented with 1% penicillin/streptomycin, 1% GlutaMAX, and 10 mM Hepes), supplemented with 1X B27 (Gibco #17504044), 10 mM Nicotinamide (Sigma #N0636), 1.25 mM N-Acetylcysteine (Sigma #A9165), 500 nM A83-01 (Tocris #2939), 1 µM Prostaglandin E2 (Tocris #2296), 0.3 µM CHIR99021 (Sigma #SML1046), 1 µM Forskolin (Tocris #1099), 50 ng/mL hEGF (Peprotech #AF-100-15), 10 ng/mL hFGF10 (Peprotech #AF-100-26), 5 ng/mL hFGF2 (Peprotech #AF-100-18B), 100 ng/mL human Noggin (Peprotech #120-10 C), and 200 ng/mL human R-spondin-1 (hRspo) (Peprotech #120-38).

For drug response assays, organoids that had been cultured for two days were harvested using 1 mg/mL Dispase (Sigma, #D4693-1G) to remove Matrigel. Organoids were subsequently washed and filtered using a 70 µM strainer to ensure uniform size, reducing variability in the assay. The filtered organoids were counted and seeded at a density of 2000 organoids per well into 96-well plates (Nunc, Thermo, #236105) using 5 µL of 95% Matrigel per well. After three days, organoids were treated with increasing concentrations of the specified drugs prepared in the expansion media using DMSO as vehicle control. Five days post-treatment, the viability of the PDOs was assessed using the CellTiter-Glo 3D assay (Promega, #G9681) by measuring luminescence using a Synergy HT plate reader (BioTek).

### Patient-derived xenografts (PDX)

Experimental protocols were performed according to the institutional guidelines of the Research Ethics and Animal Welfare Committee of the Carlos III Health Institute and the CNIO Institutional Animal Care and Use Committee (IACUC) prior to the study (Dates of approval: 4 March 2024 and 7 November 2024, approval reference: Am. 001_IACUC.003-2024 (CBA 05_2024); PROEX 073.3/24).

7–8-week-old female AthymicNude-Fox1nu mice were subcutaneously implanted with resected, and previously expanded in successive groups of mice, HNSCC tumors directly derived from patients. When the tumors were about to reach 200 mm^3^, mice were randomized into three treatment groups (six mice per group): (i) Intraperitoneal/oral vehicle (in alternate days); (ii) YKL-5-124 (10 mg/kg, 5 doses/week, intraperitoneal) and (iii) samuraciclib (70 mg/kg, 5 doses/week, orally). In the case of the 5 days experiment, mice with tumors between 200–400 mm^3^ were randomized into the same three groups, with three mice per group in this case.

Tumor size was measured twice a week with a caliper, and tumor volume was determined using the equation 0.5 × *D* × *d*^2^, where *D* is the maximum diameter and *d* is the minimum diameter. Tumor volumes for all mice in each xenograft-treatment group were averaged to obtain the mean tumor volume for the corresponding group.

Animals were sacrificed by CO_2_ asphyxiation at the end of the treatment (after 5 weeks, or after 5 days in the case of the short experiment). Tumors were resected from the flanks and measured, and the central lamina was fixed in formalin and embedded in paraffin for histological evaluation and immunohistochemistry. The rest of the tumor tissue was frozen at −80 °C. In the search of possible signs of toxicity, livers’ major lobules were also extracted, fixed in formalin and embedded in paraffin for histological evaluation. Blood extraction was also performed by intracardiac puncture and the blood cell populations were evaluated with the PE-ANIM 16 LaserCell hematological analyzer (Mindray Animal Care, #BC-5000 Vet).

### Immunohistochemistry

Paraffin-embedded PDX-derived tumor samples were cut into 3-μm sections, deparaffinized with standard xylene, and hydrated through graded alcohols into water. Antigen retrieval was performed using CC1 solution from Roche. Staining was done using a Roche Ventana Discovery Ultra IHC/ISH Slide Staining Instrument System with the primary antibodies anti-Ki-67 (30-9) rabbit monoclonal antibody (Roche Diagnostics #5278384001, ready to use) or anti-phospho-Histone H3 (Ser10) antibody (Merck Millipore #06-570, at 1:400 dilution). Then, tissue slides were counterstained with hematoxylin, mounted under coverslips, and scanned (Hamamatsu NanoZoomer-SQ). Quantification of Ki67 and pH3 staining was automatically performed using QuPath software by counting the number of positive nuclei at 20x in five independent microscopic fields per tumor section, and the mean was calculated for each treatment condition.

CDK7 staining of paraffin-embedded PDOs and PDXs (3-μm sections) was carried out using Envision Flex Target Retrieval solution, high pH (Dako, Glostrup, Denmark) on an automatic staining workstation (Dako Autostainer Plus, Glostrup, Denmark) with anti-CDK7 (MO1) mouse monoclonal (Cell Signaling Technology #2916, at 1:100 dilution) using the Dako EnVision Flex + Visualization System (Dako Autostainer, Glostrup, Denmark) and diaminobenzidine chromogen as the substrate. The final step was counterstaining with hematoxylin.

### Statistical significance

Statistical analysis was performed using GraphPad Prism version 6.0 (Graphpad Software Inc, La Jolla, CA, USA). Data is presented as the mean standard deviation (SD) of at least three independent experiments unless otherwise stated. Statistical significance was determined either using a Student’s unpaired t-test with two-tailed distribution for comparison across two groups or one-way/two-way ANOVA for comparing multiple samples/variables. In comparisons with control groups, the values of *p* < 0.05 were considered statistically significant (*p < 0.05; **p < 0.01; ***p < 0.001).

## Supplementary information

Supplementary information

Supplementary information

Supplementary information

Supplementary information

## Data Availability

RNA-sequencing data are available in the GEO repository under accession numbers GSE298508 and GSE298509.
