# Supplementary material for: CDK7-targeted therapy effectively disrupts cell cycle progression and oncogenic signaling in head and neck cancer
Source: Signal Transduct Target Ther. 2025 Nov 6;10:363. doi: 10.1038/s41392-025-02452-z (PMC12589458; doi:10.1038/s41392-025-02452-z)
Supplement: Supplementary file 1 — Supplementary information [file 41392_2025_2452_MOESM1_ESM.pdf]

Supplementary Materials for

CDK7-targeted therapy effectively disrupts cell cycle progression and oncogenic signaling in head and neck cancer

María Otero-Rosales[1,2,3]#, Miguel Álvarez-González[1,2,3], Irene Pazos[4], Beatriz de Luxán-Delgado[1,2], Sonia Del Marro[5,6], Esperanza Pozo-Agundo[1,2,3], Mar Rodríguez-Santamaría[1], Ana López-Fernández[7], Daniela Corte-Torres[8], Rocío Granda-Díaz[1,2,3], Saúl Álvarez-Teijeiro[1,2,3], Iván Fernández-Vega[8], Corina Lorz[3,5,6], Ramón García-Escudero[3,5,6], Juan P. Rodrigo[1,2,3,9], Konstantinos Tzelepis[10,11,12], George Vassiliou[10,11,13], Irene Ferrer[3,4], Mónica Álvarez-Fernández[1,2,15] ‡, Juana María García-Pedrero[1,2,3] ‡*, Francisco Hermida-Prado[1,2,3] ‡*

*Correspondence to: franjhermida@gmail.com; juanagp.finba@gmail.com

This PDF file includes:

Figures S1 to S9
Table S1
Captions for Data S1 to S3

Other Supplementary Materials for this manuscript include the following:

Data S1 to S3

- Data S1. Supplementary Data S1_HNSCC.common.dropouts.csv
- Data S2. Supplementary Data S2_DE.essential.genes.after.treatment.csv
- Data S3. Supplementary Data S3_Uncropped_WB_images.pdf

Figure S1

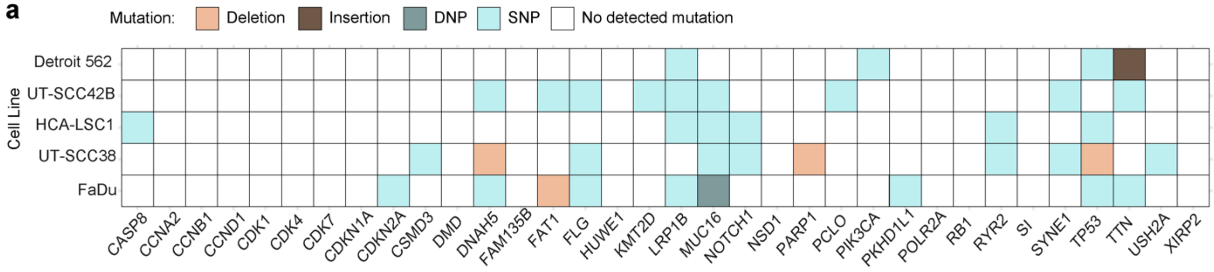

b

Name	Gender	TNM	Grade	Stage	Specimen site	Type of lesion	Primary location
FaDu	Male	-	Moderately diferentiated	-	Hypopharynx	Primary	Hypopharynx
UT-SCC38	Male	T2N0M0	Moderately diferentiated	II	Larynx	Primary	Glottic larynx
HCA-LSC1	Male	T3N2M0	Moderately diferentiated	IV	Larynx	Primary	Larynx
UT-SCC42B	Male	T4N3M0	Poorly differentiated	IV	Cervical lymph node	Metastasis	Larynx
Detroit 562	Female	M+	-	IV	Pleural fluid	Distant metastasis	Pharynx
Cal33	Male	-	Moderately diferentiated	-	Tongue	Primary	Tongue
UT-SCC2	Male	T4N1M0	Moderately diferentiated	-	Floor of the mouth	Primary	Floor of the mouth

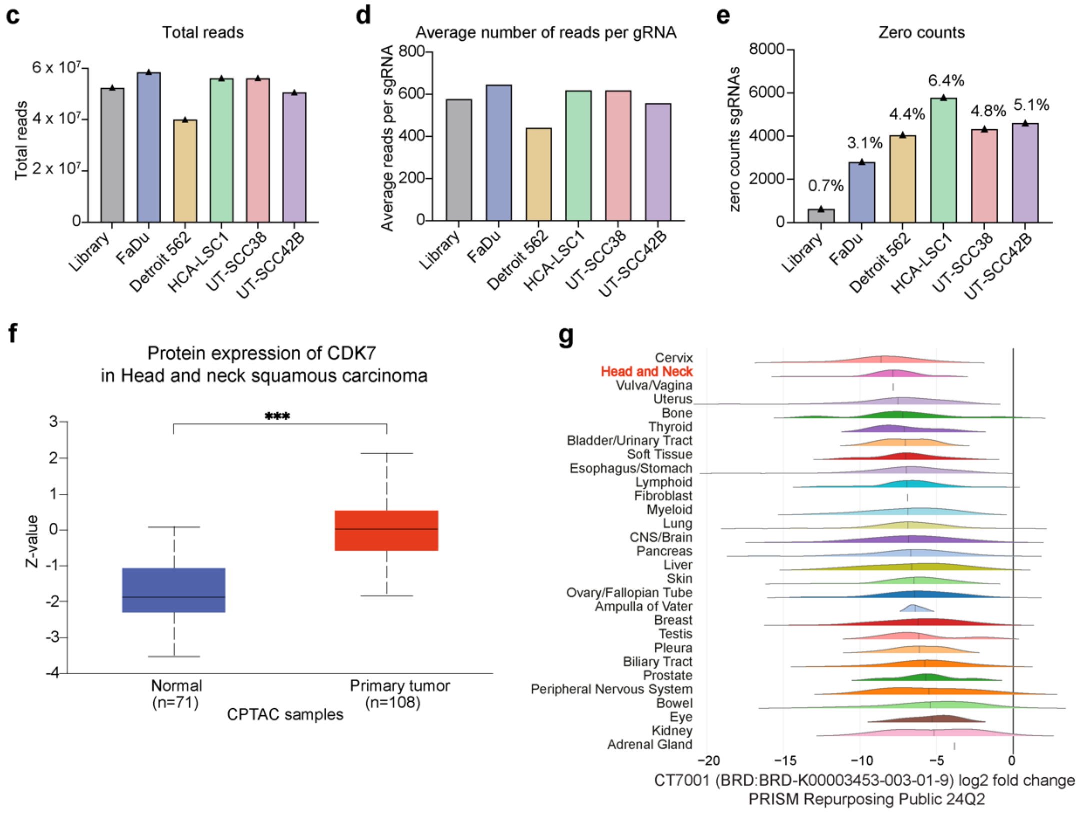

Supplementary Figure 1. CRISPR screen quality metrics and CDK7 essentiality. (a) Summary of mutations harbored by the screened HNSCC cell lines based on the most frequently mutated genes (frequency >10%) in TCGA HNSCC samples and relevant genes for this paper. **(b)** Clinical characteristics of cell line panel used in experiments. **(c)** Total number of sequencing reads per sample (corresponding to each cell line). **(d)** Average number of reads per single guide RNA (gRNA) in each sample. **(e)** Number of gRNAs with zero reads per sample. Percentages above

bars indicate the proportion of gRNAs with zero counts relative to the total number of gRNAs in the library. **(f)** CDK7 protein expression levels based on the CPTAC-HNSCC dataset for the tumor samples and the normal tissue counterparts. **(g)** Density plot of log_2 fold change values for Samuraciclib (CT7001) across cell lines from the PRISM Repurposing Public 24Q2 dataset analyzed using DepMap Data Explorer tool. Cell lines are grouped by lineage and ordered by mean log_2 fold change in ascending order.

Figure S2

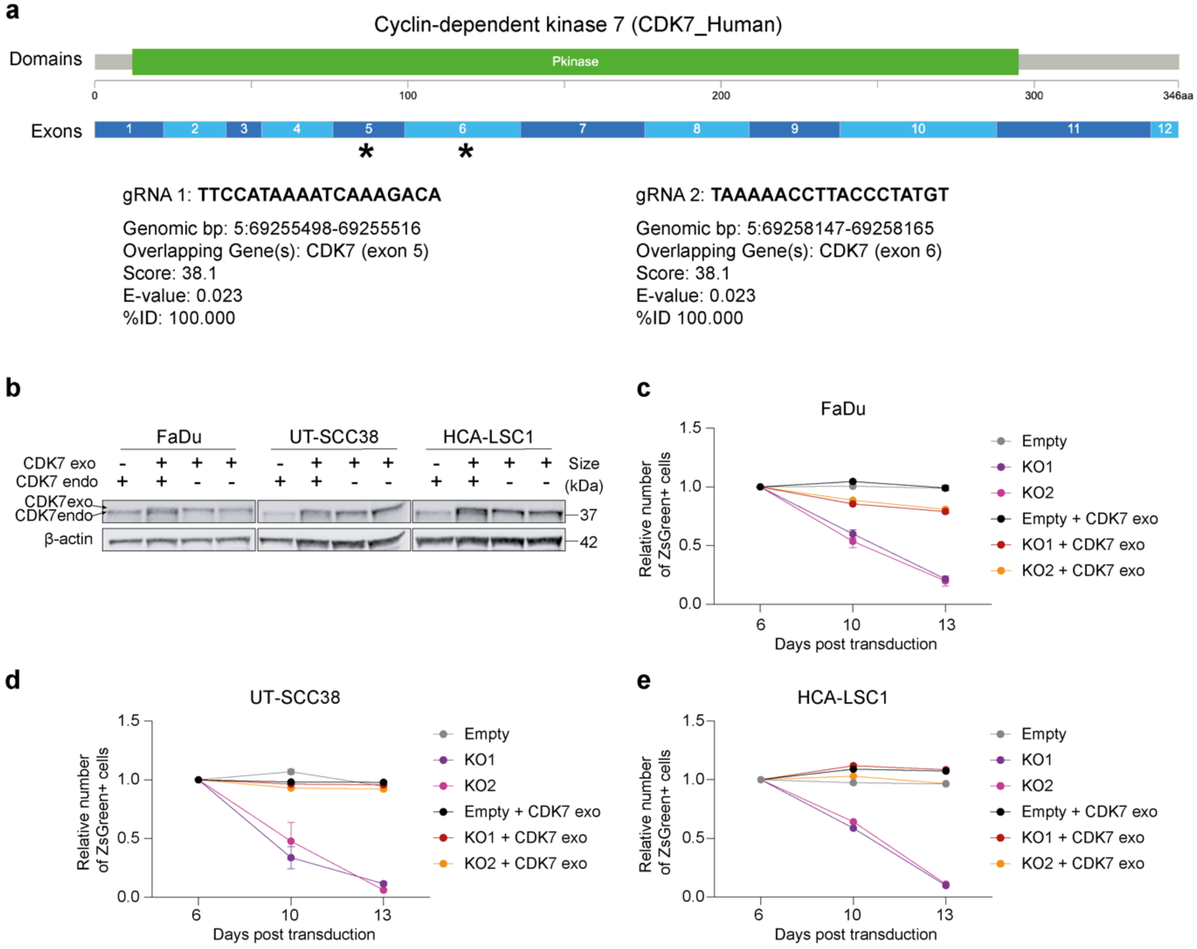

Supplementary Figure 2. CDK7-targeting sgRNAs and rescue experiment. (a) Schematic representation of CDK7 protein and exon structure, with asterisks showing the target position of the two sgRNAs used. Statistics for each sgRNA are provided, including on-target score, e-value, and sequence identity. **(b)** Western blot analysis of CDK7 protein expression in FaDu, UT-SCC38, and HCA-LSC1 cells. Endogenous and exogenous (HA-tagged) CDK7 levels were assessed in parental cells and in cells transduced with a codon-optimized, CDK7 KO-resistant construct, followed by transduction with either empty gRNA, CDK7 KO1, or CDK7 KO2 lentiviral vectors. β-actin protein serving as the loading control. **(c-e)** Line graphs comparing the effect of CDK7-targeting gRNAs (KO1 and KO2) or empty vector in parental HNSCC cell lines and their counterparts expressing a CDK7 KO-resistant construct. FaDu (**c**), UT-SCC38 (**d**), and HCA-LSC1 (**e**) cells were transduced with lentiviral vectors encoding ZsGreen (ZsG) and either CDK7-targeting gRNAs or empty vector. The percentage of ZsG-positive cells was measured by flow cytometry at multiple time points and normalized to day 6.

Figure S3

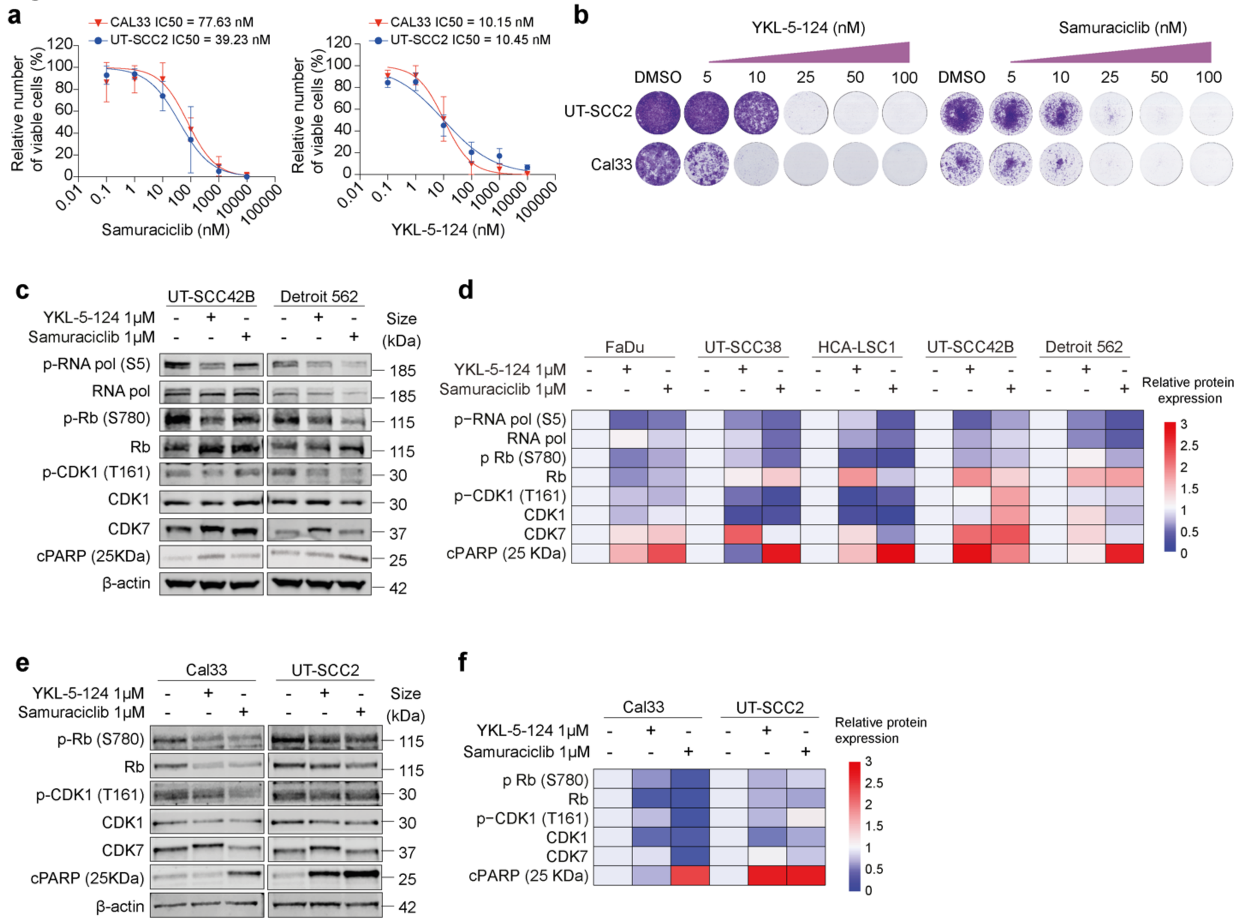

Supplementary Figure 3. Efficacy of CDK7-selective inhibitors and downstream molecular effects in HNSCC and OSCC models. (a) Measurement of cell viability in two OSCC cell lines, UT-SCC2 and Cal-33, treated with increasing concentrations of the CDK7-selective inhibitors YKL-5-124 and samuraciclib. Four replicates per condition. Data shown as average + SD. **(b)** Colony formation assays with increasing concentrations of YKL-5-124, samuraciclib in the indicated OSCC cell lines. **(c)** Western Blot analysis of CDK7 targets and cleaved PARP (cPARP) in UT-SCC42B and Detroit-562 cells treated with either vehicle (DMSO), YKL-5-124 (1µM), or samuraciclib (1µM) for 24 hours. β-actin protein was used as the loading control. **(d)** Heatmap of quantified IRDye intensities of Western blot bands in (c) and Fig. 3d for all five HNSCC cell lines, normalized to the loading control and relative to control condition. **(e)** Western Blot analysis of CDK7 targets and cleaved PARP (cPARP) in UT-SCC2 and Cal-33 OSCC cell lines. **(f)** Heatmap of quantified IRDye intensities of Western blot bands in (e) normalized to the loading control and relative to control condition.

Figure S4

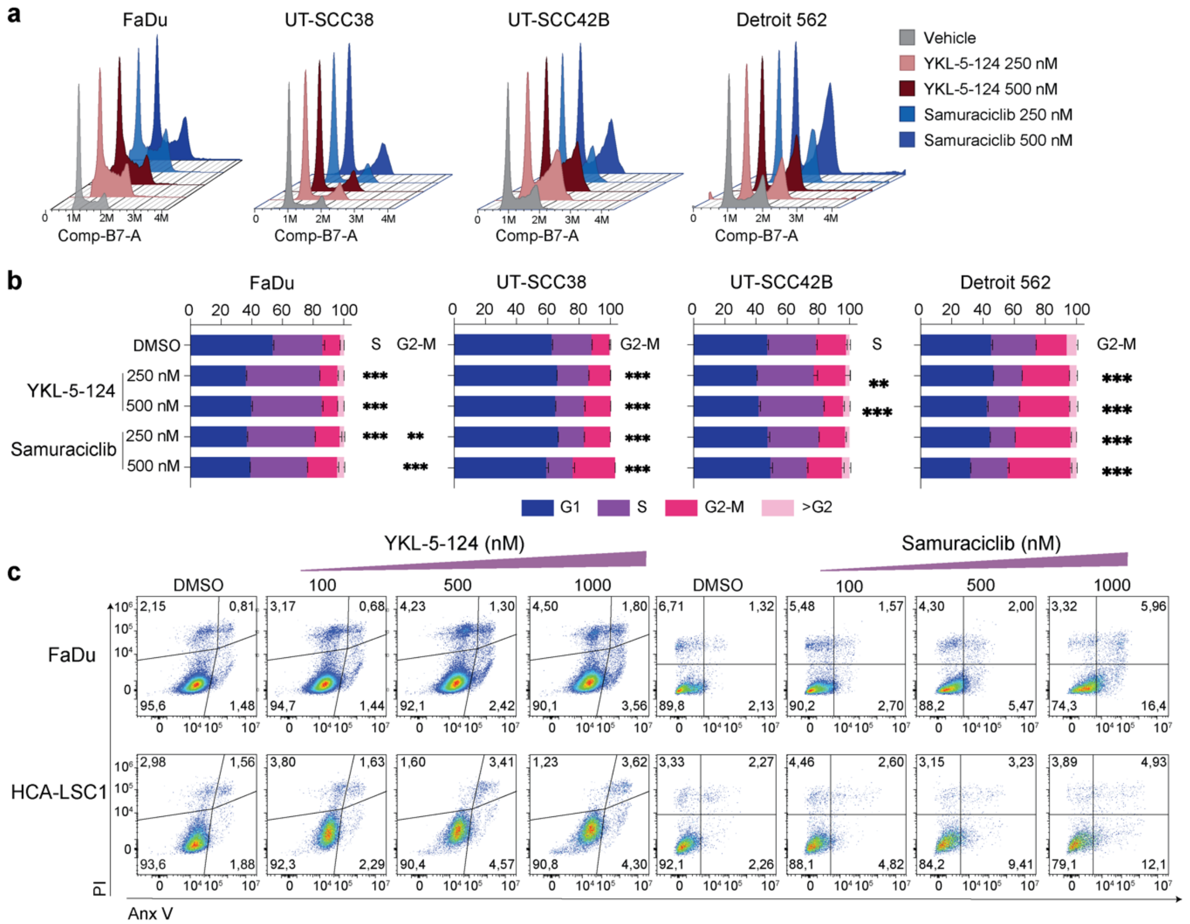

Supplementary Figure 4. **Functional characterization of CDK7 pharmacological inhibition in HNSCC cell lines. (a-b)** Flow cytometry analysis of cell cycle changes in the indicated HNSCC cell lines treated with the indicated doses of YKL-5-124 and samuraciclib for 48 hours. Only significant accumulation of cells in a cell cycle phase is indicated. Statistical significance was calculated using two-way ANOVA Dunnett's multiple comparisons test (*p < 0.05; ** p < 0.01; *** p < 0.001). **(c)** Flow cytometry dot plots of Annexin V and propidium iodide (PI) staining in FaDu and HCA-LSC1 cells treated with either vehicle or the indicated concentrations of each CDK7 inhibitor.

Figure S5

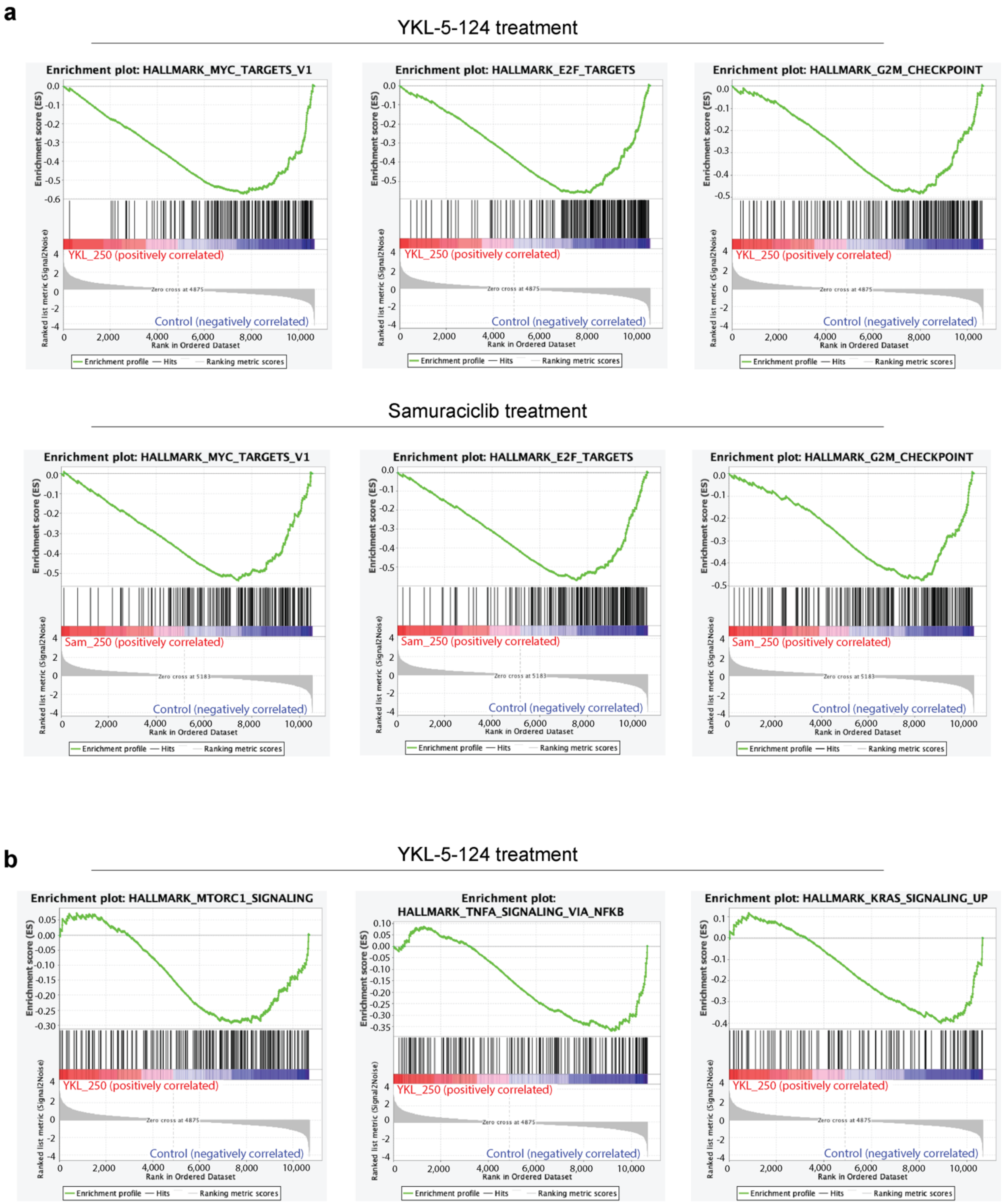

Supplementary Figure 5. Global transcriptional changes caused by selective CDK7 inhibition. (a) GSEA plots of hallmark gene sets significantly downregulated by both CDK7 inhibitors, and **(b)** gene sets specifically downregulated by YKL-5-124.

Figure S6

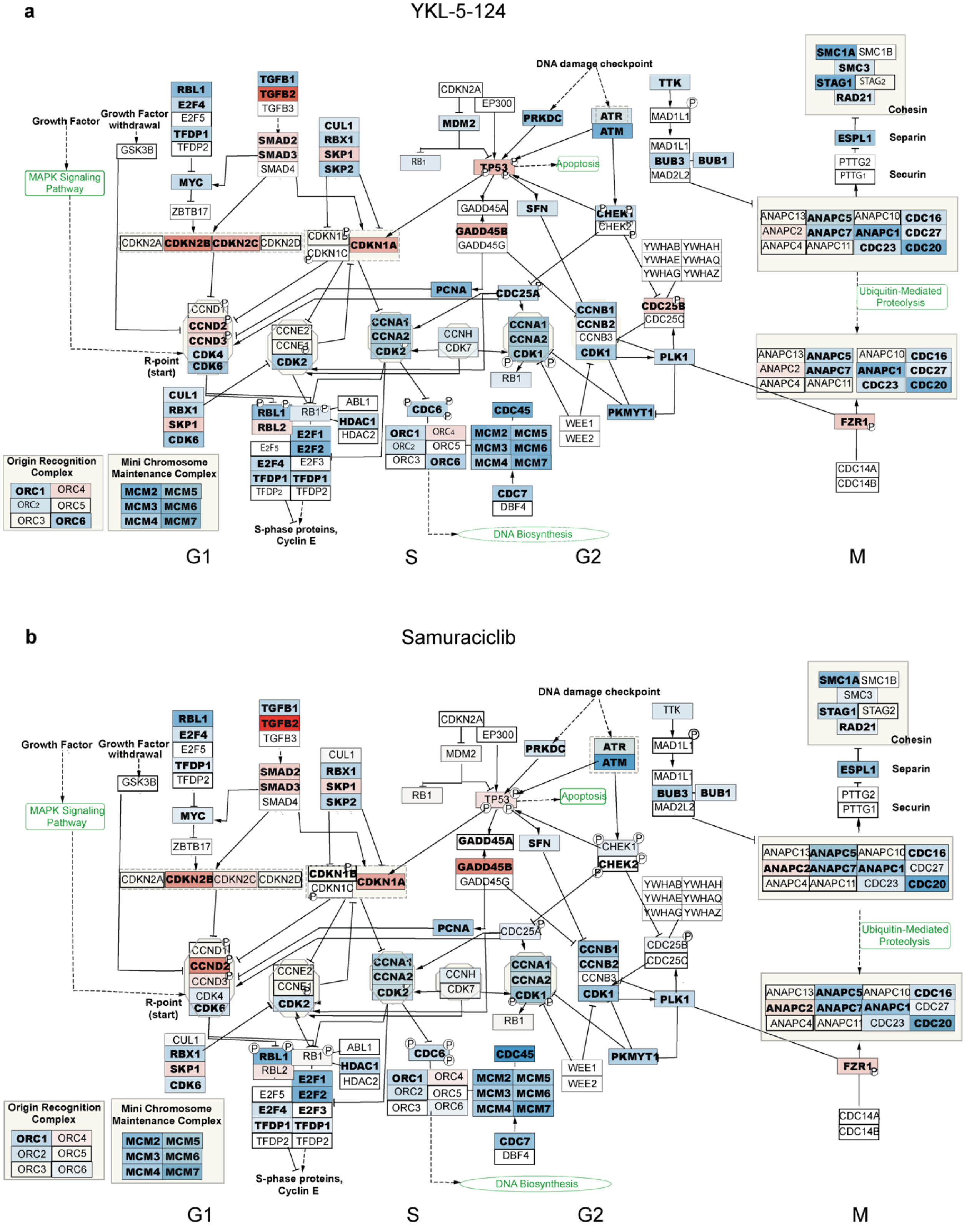

Supplementary Figure 6. Transcriptional changes in cell cycle-related genes caused by selective CDK7 inhibition. (a-b) Schematic representation of cell cycle pathway genes colored by differential expression levels upon CDK7 inhibition with YKL-5-124 **(a)** and samuraciclib **(b)**.

Downregulated genes are shown in blue and upregulated genes in red. Significant changes are highlighted in bold.

Figure S7

a

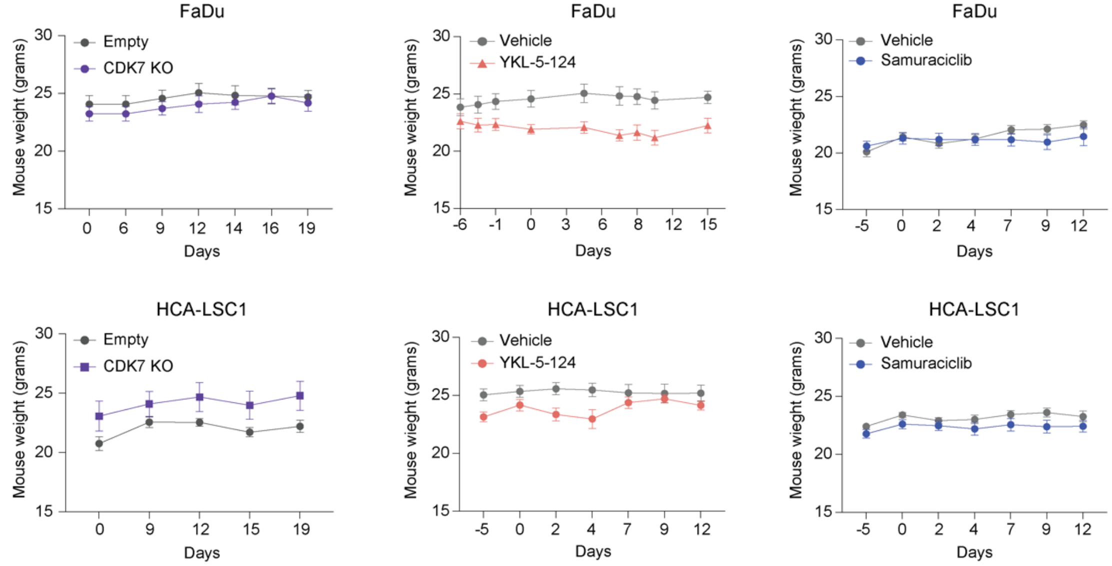

Supplementary Figure 7. Impact of CDK7 inhibition on mice body weight. (a) Mice whole-body weight along each experiment and condition. No significant differences were observed between groups, Two-way ANOVA test: n.s., non-significant.

Figure S8

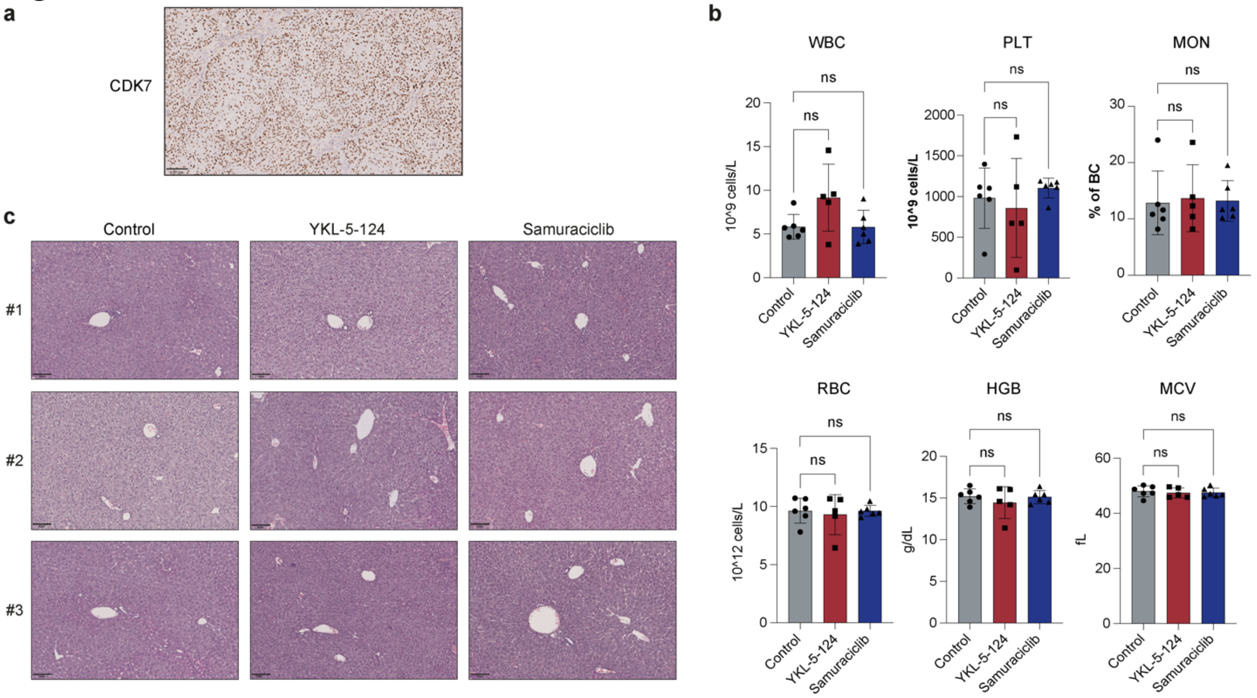

Supplementary Figure 8. CDK7 expression and *in vivo* toxicity assessment of CDK7 inhibitors using a HNSCC PDX model. **(a)** Representative immunohistochemistry image of CDK7 staining in the PDX model (scale bar, 100 µm). **(b)** Hematological analysis of PDX mice after 5 weeks of treatment with vehicle (control), YKL-5-124, or samuraciclib. Blood parameters include white blood cells (WBC), platelets (PLT), monocytes (MON), red blood cells (RBC), hemoglobin (HGB), and mean corpuscular volume (MCV). Data are represented as mean ± SD. Statistical significance was assessed by one-way ANOVA: n.s., non-significant. **(c)** Representative H&E-stained liver sections from three animals per group (control, YKL-5-124 and samuraciclib), showing no histopathological abnormalities (scale bar, 100 µm).

Figure S9

a

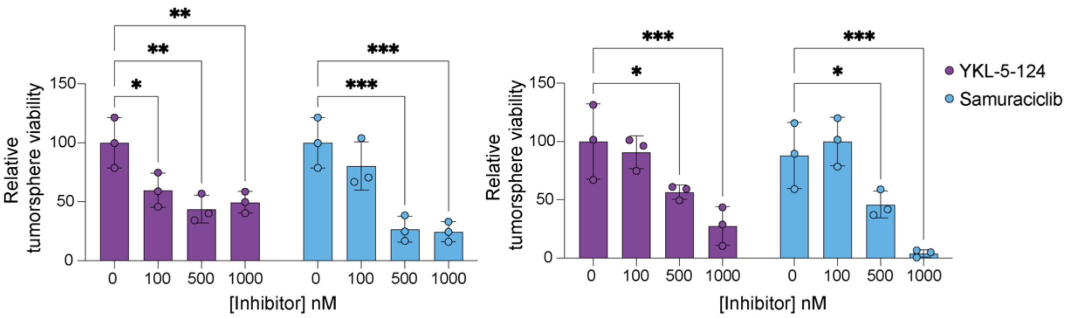

Supplementary Figure 9. Impact of CDK7 inhibition on HNSCC tumorspheres. (a) Measurement of viability of HNSCC-derived tumorspheres grown for 7 days and treated with increasing concentrations of YKL-5-124 and samuraciclib in FaDu and UT-SCC38 cells for 5 days. Each condition was performed in triplicates (Two-way Anova, *$p < 0.05$; ** $p < 0.01$; *** $p < 0.001$).

Table S1. Primary antibodies used in Western blot.

Primary antibody	Supplier (#Cat No.)	Protein MW (kDa)	Host specie	Dilution
Anti-CDK7	Cell Signaling (#2916)	40	Mouse	1/1,000
Anti-p-Ser5-RNA Pol II	Cell Signaling (#13523T)	250	Rabbit	1/1,000
Anti-RNA Pol II	Cell Signaling (#14958T)	250	Rabbit	1/1,000
Anti-p-Rb	Cell Signaling (#9307S)	110	Rabbit	1/1,000
Anti-Rb	Cell Signaling (#9309S)	110	Mouse	1/1,000
Anti-β-actin	Sigma (#A2228)	42	Mouse	1/1,000
Anti-CDK7	Cell Signaling (#2916)	40	Mouse	1/1,000
Anti-p-CDK1	Cell Signaling (#9114S)	34	Rabbit	1/1,000
Anti-cdc2/CDK1	Cell Signaling (#9116)	34	Mouse	1/1,000
Anti-Cleaved-PARP	Abcam (#ab32064)	25	Rabbit	1/1,000
Anti-β-actin	Sigma (#A2228)	42	Mouse	1/10,000

Data S1. (separate file)

Excel table with essential genes common to all five HNSCC cell lines screened with their corresponding negative false discovery rate (FDR) values.

Data S2. (separate file)

Excel table of genes listed in Data S1 with significant differential expression levels (as log2FoldChange) at least in one condition.

Data S3. (separate file)

Uncropped images of Western blot membranes.
