# Supplementary material for: CDK7-targeted therapy effectively disrupts cell cycle progression and oncogenic signaling in head and neck cancer
Source: Signal Transduct Target Ther. 2025 Nov 6;10:363. doi: 10.1038/s41392-025-02452-z (PMC12589458; doi:10.1038/s41392-025-02452-z)
Supplement: Supplementary file 4 — Supplementary information [file 41392_2025_2452_MOESM4_ESM.pdf]

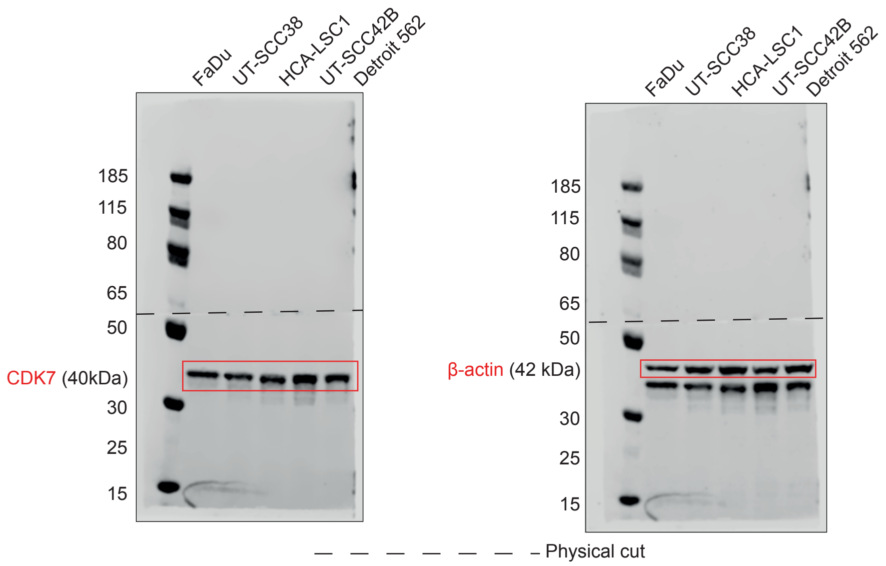

Uncropped membranes for Figure 2a

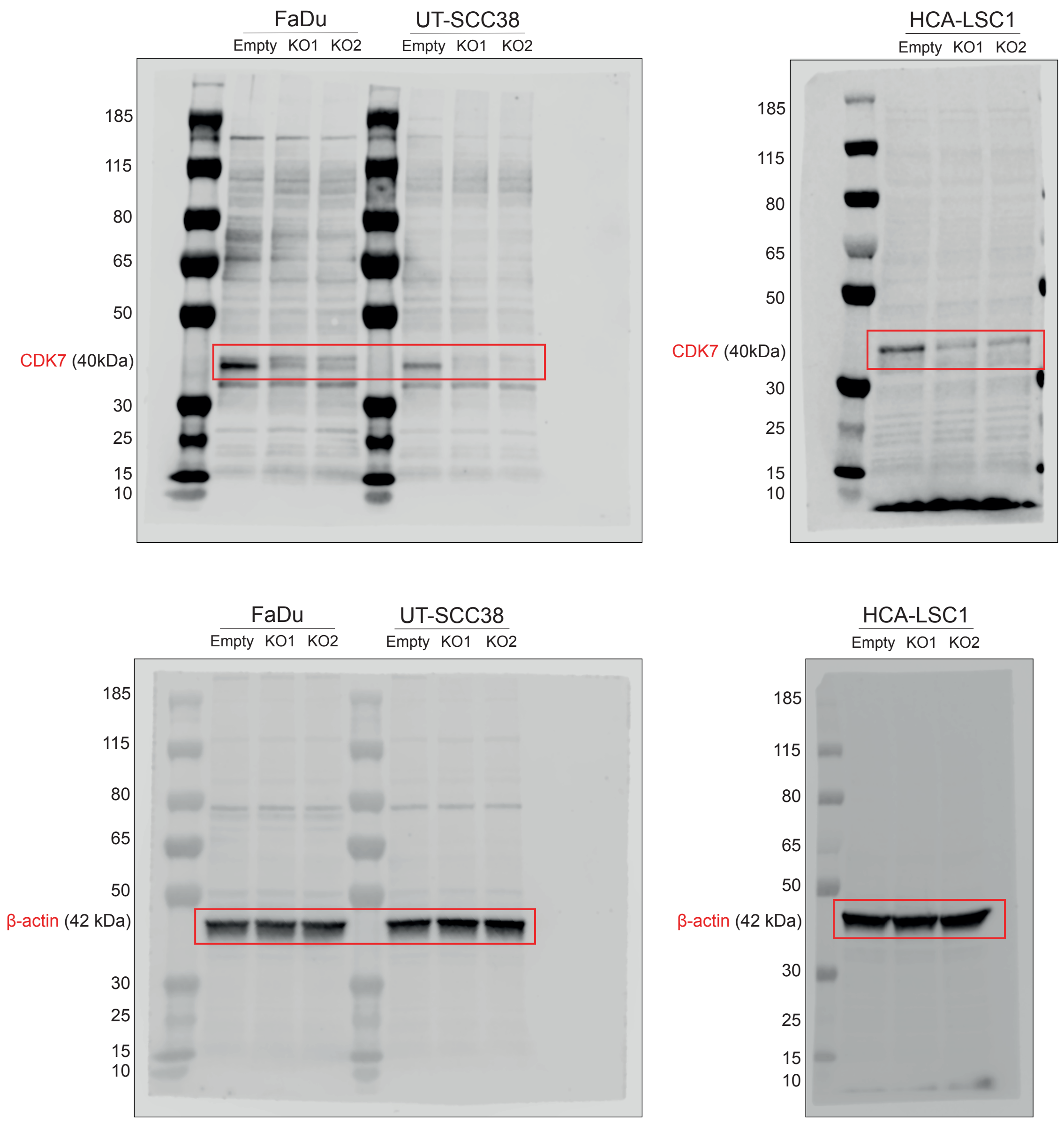

Uncropped membranes for Figure 2b

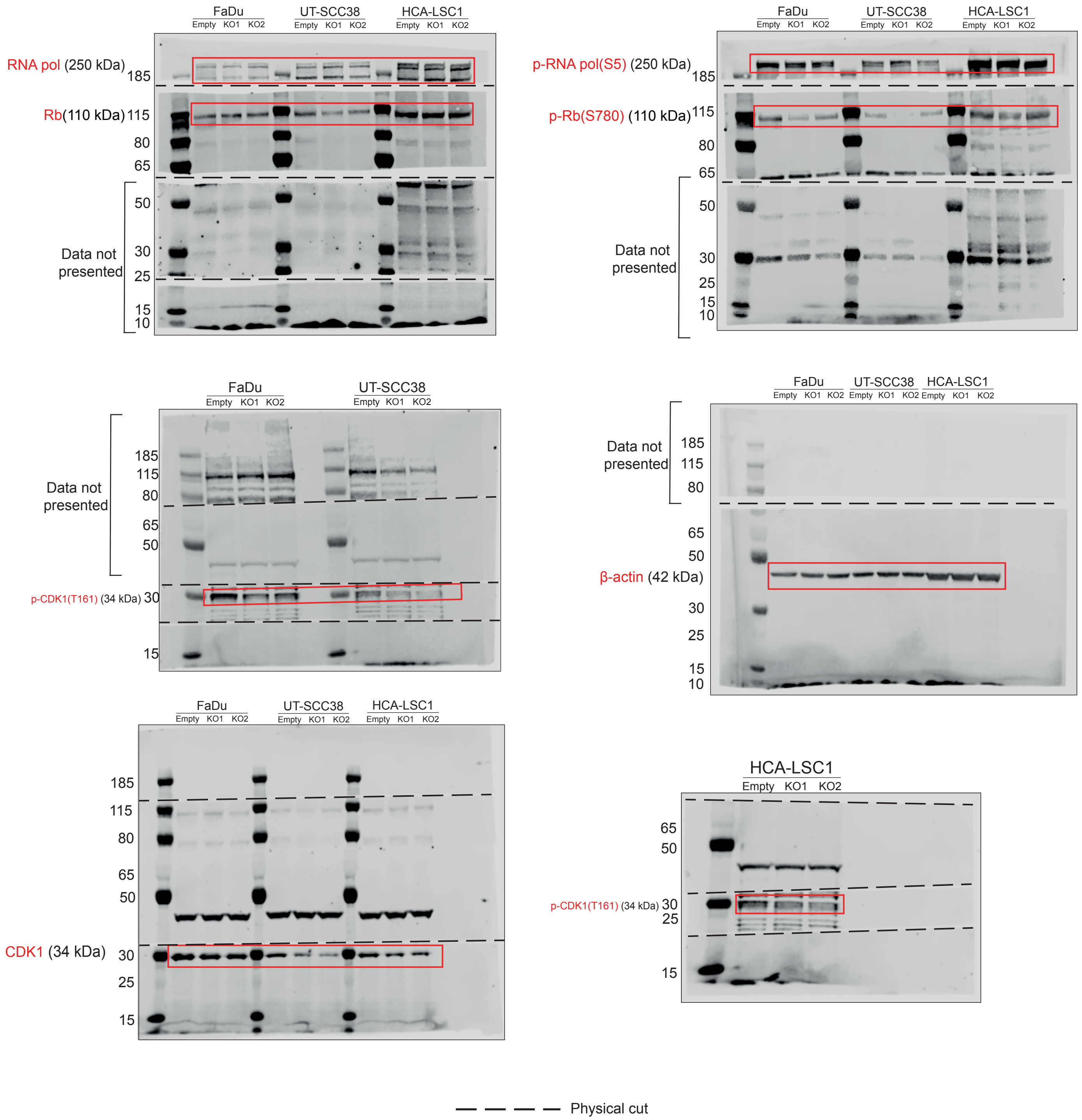

Uncropped membranes for Figure 2g

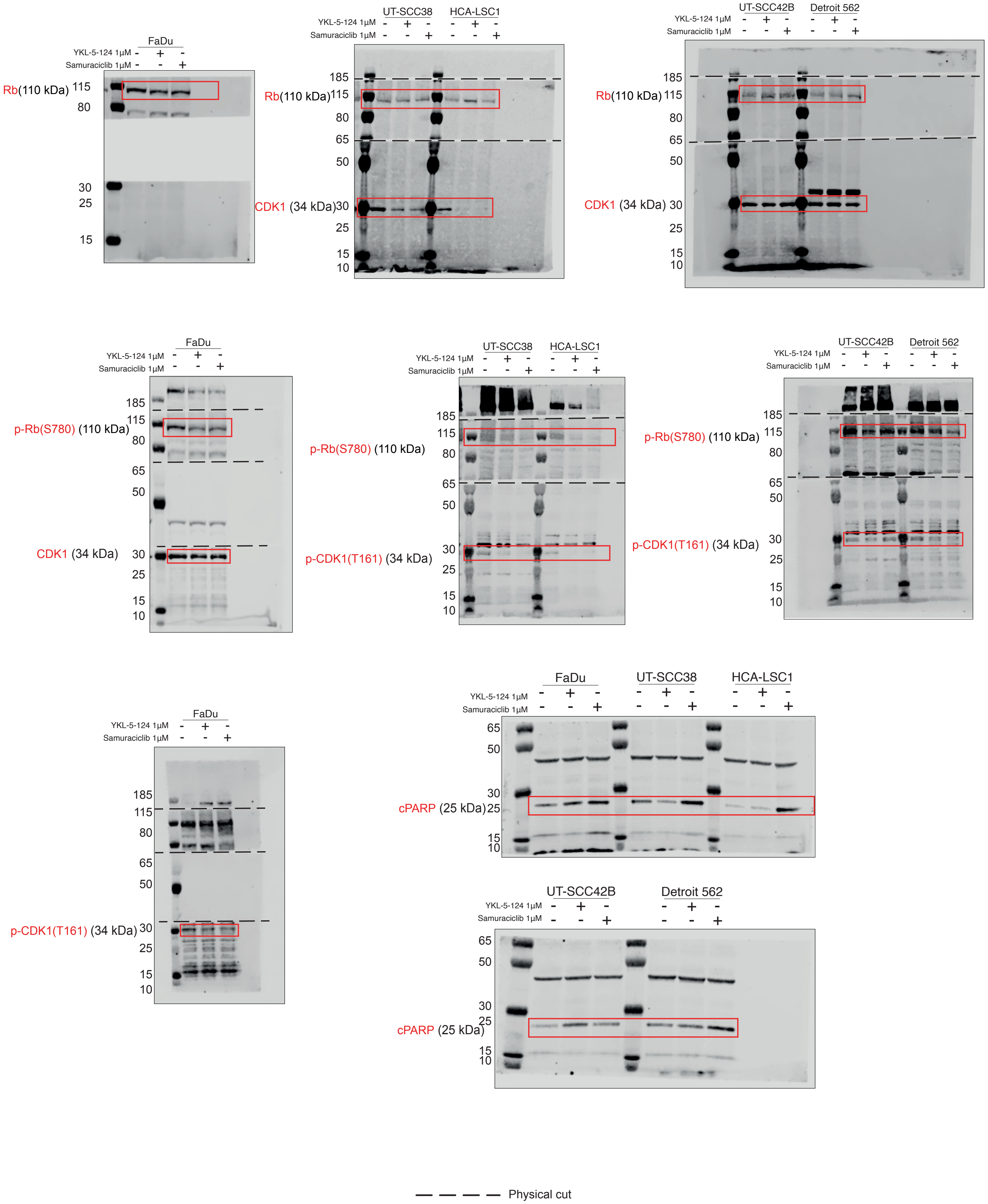

Uncropped membranes for Figure 3d and Supplementary Figure 1a (1)

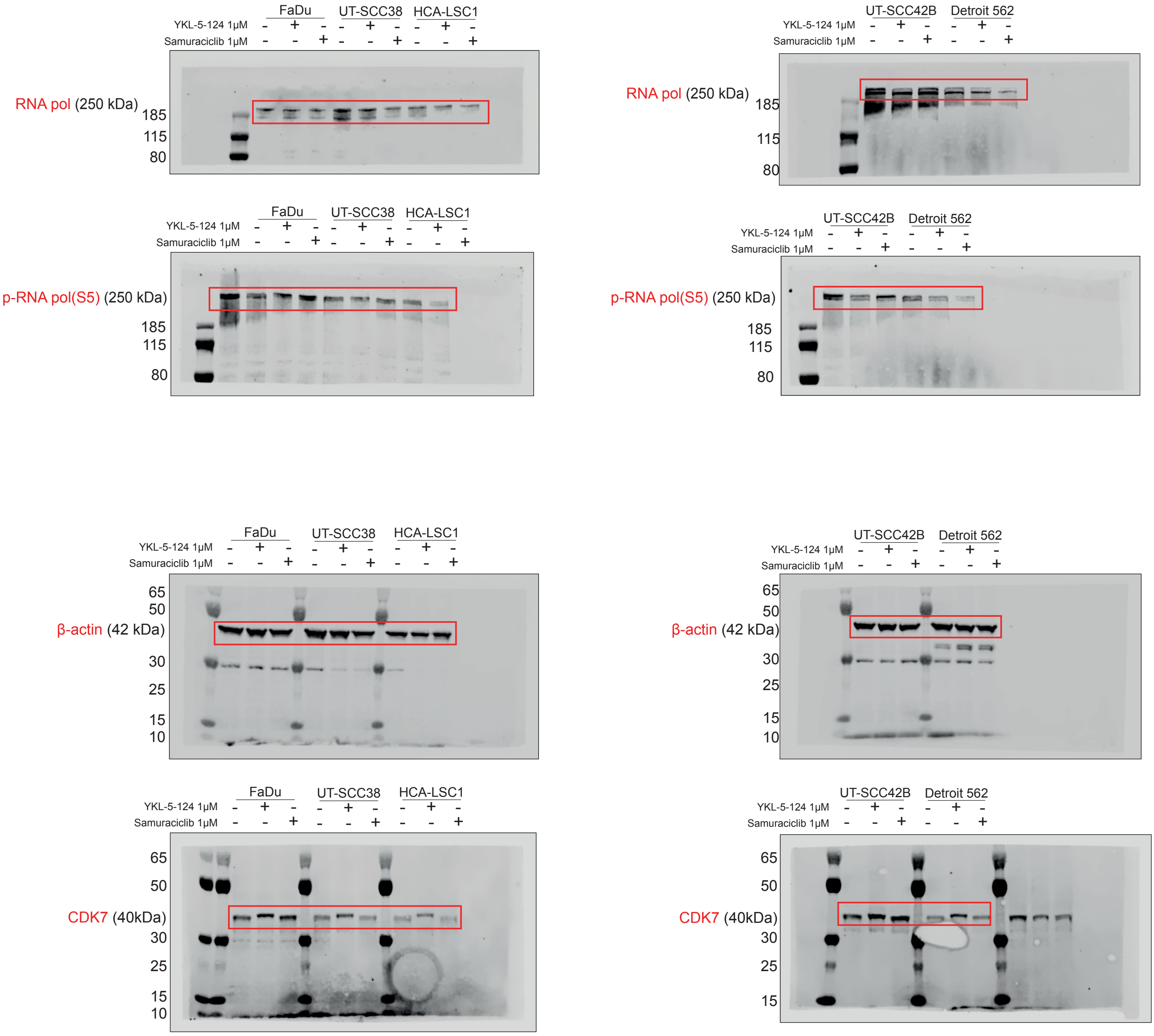

Uncropped membranes for Figure 3d and Supplementary Figure 1a (2)

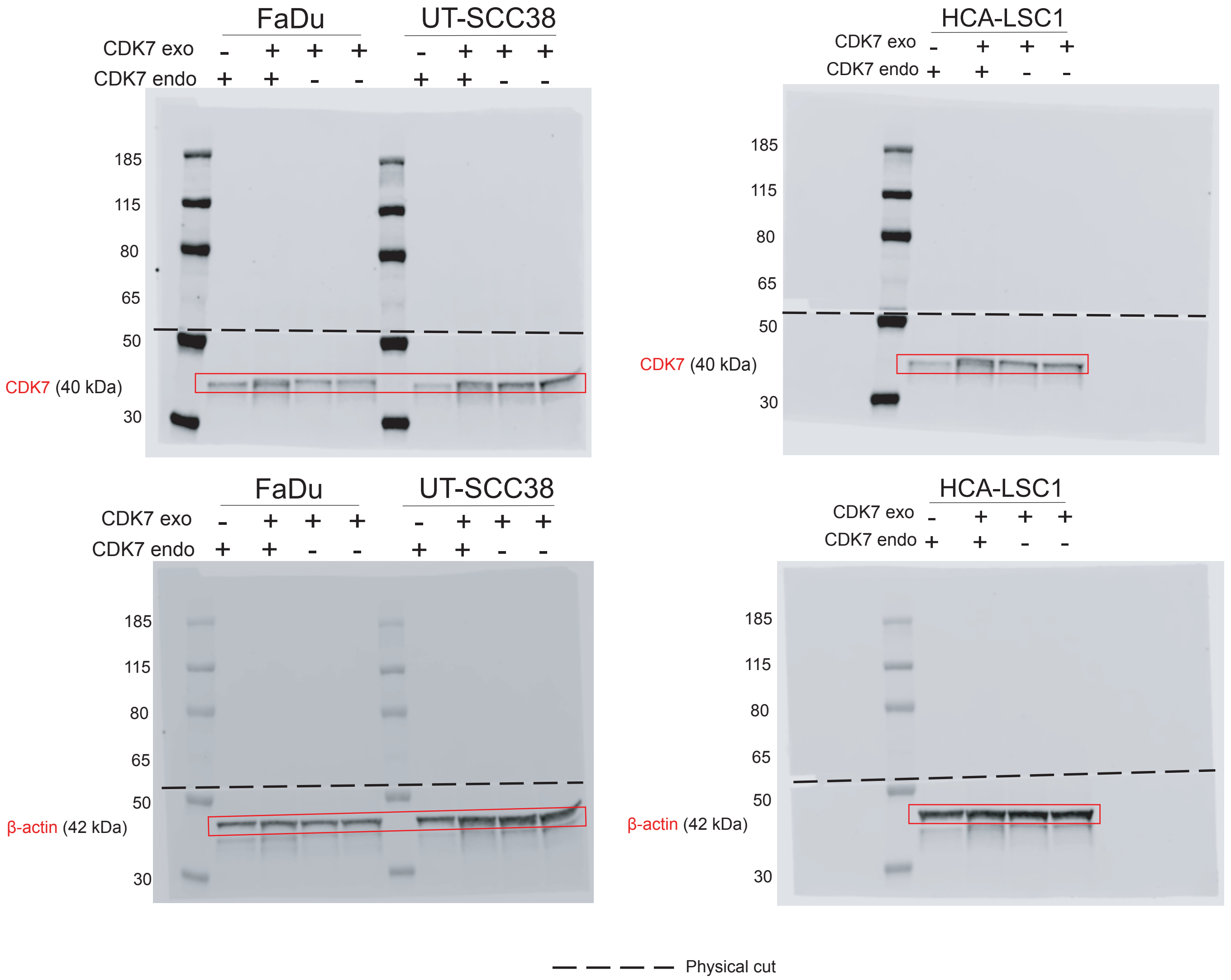

Uncropped membranes for supplementary figure 2b

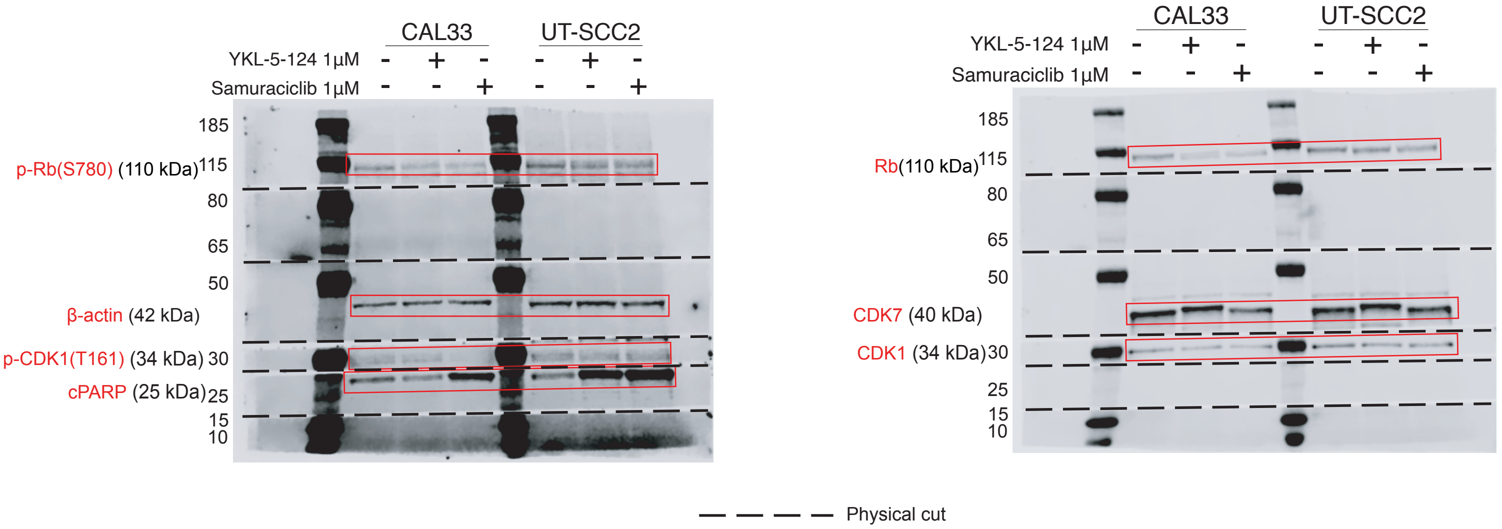

Uncropped membranes for supplementary figure 3e
